# Understanding Transformer-Based Classifications of Medical Text Using a Large Language Model for the Attribution of Feature Importance: Proof-of-Concept Algorithm Development and Validation Study

**DOI:** 10.2196/81644

**Published:** 2026-06-10

**Authors:** Fangwen Zhou, Ashirbani Saha, Muhammad Afzal, Rick Parrish, R Brian Haynes, Alfonso Iorio, Cynthia Lokker

**Affiliations:** 1 Health Information Research Unit, Department of Health Research Methods, Evidence, and Impact, Faculty of Health Sciences, McMaster University, 1280 Main Street West, Hamilton, ON, L8S 4L8, Canada, 1 905-525-9140 ext 22208; 2 Department of Oncology, Faculty of Health Sciences, McMaster University, Hamilton, ON, Canada; 3 Department of Computer Science, Birmingham City University, Birmingham, United Kingdom; 4 Department of Medicine, Faculty of Health Sciences, McMaster University, Hamilton, ON, Canada

**Keywords:** artificial intelligence, explainable artificial intelligence, feature attribution, integrated gradients, Shapley Additive Explanations, SHAP, GPT, deep learning, natural language processing

## Abstract

**Background:**

Deep learning, particularly encoder-only transformer architectures, has demonstrated excellent performance in biomedical literature classification, facilitating evidence-based medicine, and knowledge synthesis. However, the opacity of these models’ decision-making processes limits their clinical interpretability, trustworthiness, and widespread adoption. Traditional explainable artificial intelligence methods, such as Shapley Additive Explanations (SHAP) and integrated gradients (IG), address this issue but often incur substantial computational overhead for text classification. Generative large language models may offer a novel approach to generating interpretable, context-aware explanations as autonomous agents.

**Objective:**

As a proof-of-concept, the study aimed to investigate the effectiveness of GPT-4o as a standalone, end-to-end perturbation-based explainer for a BioLinkBERT text classifier. We compared its explanations against the SHAP partition explainer and IG as established baselines in terms of explanation faithfulness and semantic alignment.

**Methods:**

A stratified sample of 200 studies from the McMaster Premium Literature Service (PLUS) and Clinical Hedges databases was classified by a fine-tuned BioLinkBERT model for methodological rigor. The sampling specifically over-represented difficult, low-confidence predictions to rigorously test the explainers, with an equal number of studies sampled from each probability decile predicted by BioLinkBERT. GPT-4o, SHAP, and IG generated token-level feature attributions across a robust feature space of 80,901 tokens. GPT-based explanations were derived through a sophisticated, iterative masking perturbation workflow under 2 prompting schemes (token indices vs explicit subword tokens). Explanations were evaluated using a rank-based, modified area over the perturbation curve (AOPC), pairwise correlation analyses, and qualitative assessment of feature importance.

**Results:**

Among the 200 studies, 80,901 tokens were included, and feature attributions were generated by the 4 explainers (6369 unique tokens). SHAP (AOPC 0.222, 95% CI 0.200-0.244) and IG (AOPC 0.225, 95% CI 0.202-0.247) provided consistent explanations, effectively identifying tokens relevant to study rigor (eg, “randomized” and “blind”). In contrast, despite evaluating a larger perturbation space, the GPT-4o prompting schemes did not achieve comparable faithfulness (AOPC 0.025-0.029) and produced divergent token attributions. Correlation analysis demonstrated moderate alignment between SHAP and IG (Pearson *r=*0.367), whereas GPT-4o exhibited limited correlation (Pearson *r*≤0.032) with the established baselines. Sensitivity analyses isolating only correctly classified instances yielded similar trends. Additionally, the iterative application programming interface calls required for GPT made it significantly more computationally intensive and costly to execute, whereas IG was the most temporally efficient.

**Conclusions:**

Despite their advanced contextual capabilities, current generative large language models are limited when deployed as standalone perturbation explainers. The findings reveal that GPT-4o struggles to accurately synthesize mathematical feature importance through iterative masking, lacking the reliability of traditional explainable artificial intelligence frameworks. Future research could build upon this work and investigate specialized prompt engineering, whole-word recombination strategies, and hybrid frameworks.

## Introduction

The rapid growth of biomedical literature has driven the development of automated classification systems to facilitate knowledge synthesis and translation [1]. Deep learning, particularly encoder-only transformer architectures such as Bidirectional Encoder Representations from Transformers (BERT), has gained significant attention in biomedical text classification [2]. These models excel due to their ability to capture contextual information, leverage transfer learning, and minimize the need for extensive data preprocessing and feature engineering, making them highly effective for biomedical applications [3,4].

However, the complex, multilayered nature of BERT models undermines their interpretability, posing challenges in understanding their decision-making processes [5]. Explainable artificial intelligence (XAI) techniques aim to address this limitation by providing insights into feature importance [6]. One widely used XAI framework is Shapley Additive Explanations (SHAP), which is grounded in game theory and uses Shapley values to systematically estimate feature contributions by perturbing inputs [7]. Despite its theoretical robustness, SHAP has substantial computational overhead. It requires summing marginal contributions across feature subsets, which leads to an exponential increase in complexity as the feature space grows [8]. Consequently, computing SHAP values becomes impractical for BERT models that process long sequences of up to 512 tokens.

To mitigate this challenge, a partition explainer groups features into structured partitions, which reduces complexity while preserving interactions. By approximating Shapley values using Owen values [9], the partition explainer enhances scalability, making it particularly suitable for high-dimensional text classification tasks. Another widely used method is integrated gradients (IG) based on the Aumann-Shapley method, which ensures axiomatic fairness and path-integrated attribution of feature importance [10-14]. It offers a computationally efficient approach to estimating feature importance by measuring the accumulated gradients along the path between the baseline input and the instance input. IG has been widely applied in natural language processing (NLP) tasks, providing a balance between interpretability and computational feasibility [10-12]. However, these methods face challenges in explaining text classifiers due to significant multicollinearity between input tokens and high-dimensional feature spaces [10,15,16].

More recently, pretrained generative large language models (LLMs) leveraging transformer decoders have garnered wide attention in NLP due to their performance and flexibility [17]. Previous studies, such as those by Zytek et al [18,19] and Zeng and Zhu [20], explored LLMs in model explanation, investigating the use of LLMs to convert SHAP explanations into plain-text descriptions to improve human interpretability. Unlike perturbation-based XAI methods or gradient-based XAI methods, LLMs can generate explanations while incorporating token-level contextual relationships, potentially leading to more faithful feature attributions. More recently, LLMs have started to support structured JSON output and function calling, providing a convenient way to integrate model predictions [21-23].

Despite these advances, no prior studies have explored the usage of LLMs as standalone explainers for deep learning models in biomedical text classification. To address this gap, as a proof-of-concept, we develop and validate a methodology to investigate GPT-4o by OpenAI, as an end-to-end, agentic perturbation explainer for a BERT-based biomedical text classifier. We compare its performance against SHAP’s partition explainer and IG explanations.

## Methods

### Classifier and Dataset Description

This study builds upon the work from a previous study [24], where we fine-tuned 630 encoder-only transformer models using grid search. The data came from the McMaster Premium Literature Service (PLUS) and the Clinical Hedges databases associated with the McMaster Health Information Research Unit. Detailed descriptions of these 2 databases are published elsewhere [24-28]. In short, both databases include treatment, primary prevention, and/or quality improvement studies that had been manually appraised using custom criteria for randomized controlled trials [29] to determine whether they were methodologically rigorous or nonrigorous. Studies in the PLUS database from inception (2003-2023; n=53,219) were used for training (n=42,575), validation (n=5322), and testing (n=5322). Studies from 2024 in McMaster PLUS (n=1011) and the Clinical Hedges (n=6572) were used for external testing. The top-performing models were identified on the validation set and subsequently tested.

For this study, we selected a stratified random sample of 200 studies, 40 from each dataset. For each of the 5 data subsets, studies were placed into 10 bins based on their predicted probability for rigor, and a random sample of 4 studies per probability bin per dataset was selected. The probability scores were generated by the model that had the lowest validation loss, which was a BioLinkBERT-based model with a learning rate of 3 × 10^5^, a batch size of 64, a random seed of 2, and included class weight adjustments. The model was fine-tuned for 5 epochs before premature termination by early stopping, and weights from epoch 2 were used as it achieved the lowest validation loss. Other relevant configurations can be found in our previous publication [26]. The model achieved a cross-entropy loss of 0.291, an area under the receiver-operating characteristic curve of 0.941, and an accuracy of 0.879 on the full validation set.

### SHAP Explanations

We used the SHAP partition explainer [30] to compute an Owen value for each token in each prediction. The partition explainer was chosen due to its efficiency in high-dimensional text classification and its ability to capture feature interactions more effectively than standard Shapley value approximations [31]. SHAP values were calculated using logits that were back-transformed from SoftMax probabilities.

### IG Explanations

We used IG to estimate token-level feature attributions for each prediction. We used an empty sequence padded to 512 tokens with “[PAD]” as the baseline input, ensuring the absence of semantic content while preserving the tokenization structure. The baseline input produces a rigorous probability of 11.7%. Attributions were derived by computing gradients with respect to the input embeddings across 30 interpolation steps. The total IG attribution per token was calculated by aggregating gradients across all embedding dimensions.

### GPT Explanations

#### Overview

**Figure 1.**
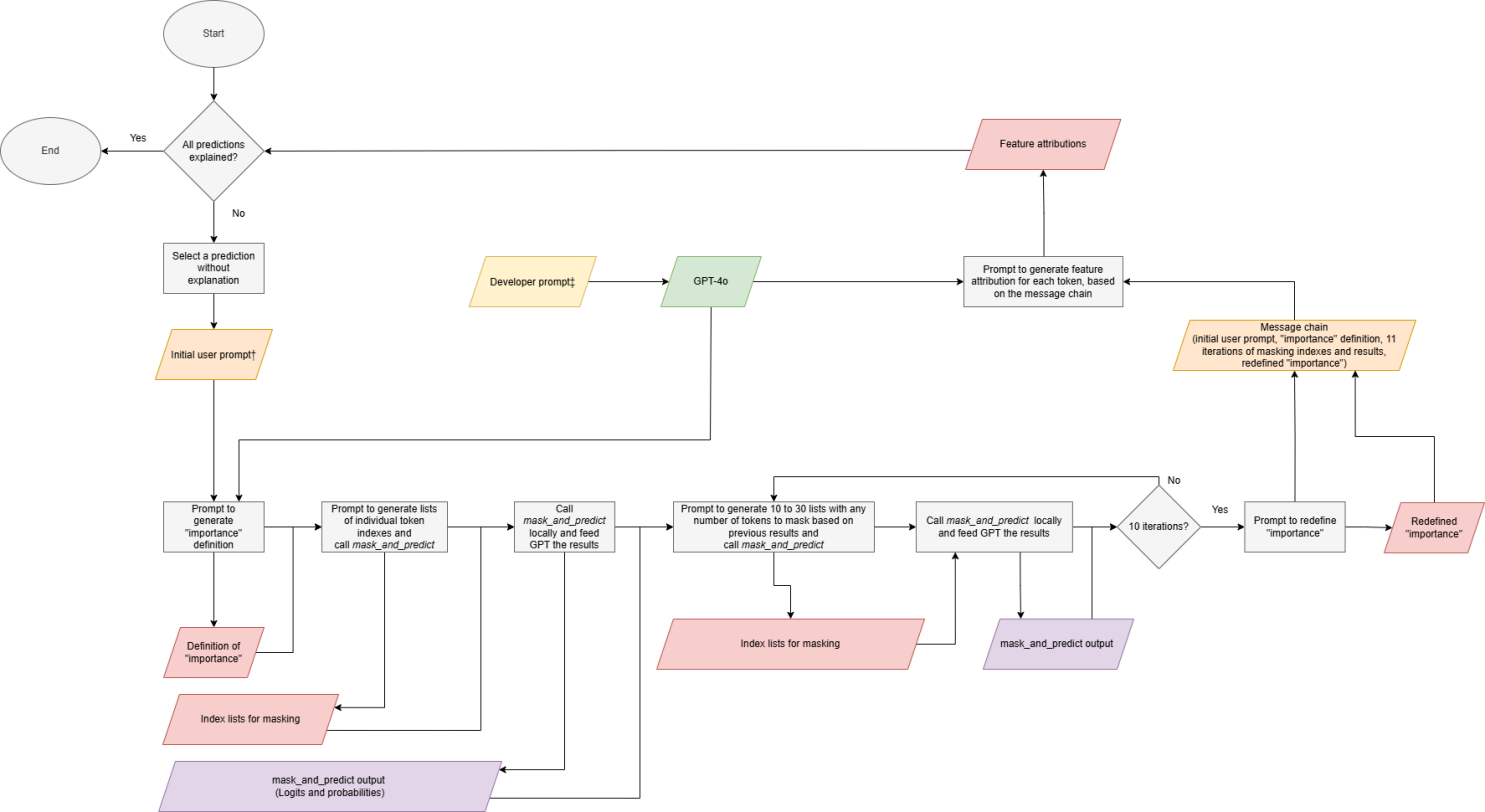
Flowchart of the pipeline for generating explanations and feature attributions from GPT-index and GPT-token. The pipeline illustrates the iterative perturbation-based workflow, including the developer prompt, the initial user prompt, repeated masking iterations, and batched feature attribution generation using structured outputs. †Input tokens are included only in the initial user prompt for GPT-token. ‡The information and instructions provided in the developer prompt differ for GPT-index and GPT-token, as GPT-index was not provided with the input tokens. Detailed prompts can be found in Multimedia Appendix 1.

We used GPT-4o-2024-11-20 with a temperature of 0, and both presence and frequency penalties set to 0, to ensure deterministic outputs. The objective was to evaluate GPT’s ability to estimate token-level feature attributions through perturbation-based explanations, similar to SHAP. A total of 2 prompting schemes, GPT-index and GPT-token, were designed to systematically mask tokens and assess their influence on classifier predictions. Tokens were obtained by processing the original input through BioLinkBERT’s word-piece tokenizer. Both schemes received the number of input tokens, predicted logits for both classes, and the probability of the positive class. Additionally, GPT-token was provided with the complete list of input tokens in a comma-separated format and the manual appraisal criteria. The full prompts used for both schemes are available in Tables S1-S3 in Multimedia Appendix 1. A flow diagram can be found in Figure 1.

#### Developer Prompt

In the developer prompt, GPT was provided with (1) the role of a machine learning model explainer, (2) the task of explaining a binary encoder-only transformer text classifier’s prediction via perturbations by masking input tokens, (3) the scheme-specific information that would be provided in the user prompts, and (4) step-by-step instructions on defining importance, masking, function calling, and generating importance values that would be executed subsequently. The manual appraisal criteria [31] for GPT-tokens were included in the developer prompt.

#### Initial User Prompt

In the initial user prompt, both prompting schemes (GPT-index and GPT-token) were provided with the number of tokens, the predicted logits of the positive and negative classes, and the probability of the positive class. The input tokens, in the format of a comma-separated list, were provided to GPT-token only in the initial user prompt.

#### Subsequent User Prompts

The model was first instructed to generate the definition of “importance” for itself and then to call *mask_and_predict* with lists of individual indices (eg, [[0] [1], … [x-1]], for an input with x tokens), echoing the instructions provided in the developer prompt. To call *mask_and_predict*, we used the function-calling feature [32] in OpenAI’s application programming interface (API). The function, in general, takes lists of integers as input and returns the logits for both classes and the probability of the positive class for each list of indices to mask, with every token at the index replaced with “[MASK].”

Subsequently, the model was prompted 10 times to generate 10 to 30 lists with any number of indices to mask and call *mask_and_predict*, where each iteration included the results of all previous iterations. The model was explicitly instructed to avoid generating the same combinations of indices and to adapt future masking based on prior iteration results. Finally, the model was asked to redefine “importance” based on the initial definition and the results of all masking iterations.

#### Feature Attribution Calculations

The model was prompted, with the final message chain including the initial user prompt, all iterations of perturbations, and both iterations of importance definition, to generate the feature importance for each token, 20 tokens per batch. Crucially, while the output generation was batched to bypass GPT’s limitations in generating long structured sequences, the full global context of all masking permutations and model predictions was retained in the prompt for every batch. The model was not provided with the calculated feature attributions of other batches, as the mathematical calculation based on its own definition only required the global perturbation history, which was always present. This batched approach was taken because the model often had issues with generating longer sequences. The structured output function [33] of the API was leveraged to generate a list of dictionaries of token indices and their corresponding feature attributions.

### Evaluation

#### Area Over the Perturbation Curve

To establish feature attribution performance, we used a modified version of the area over the perturbation curve (AOPC), which was used in previous literature [16,34,35]. The AOPC was calculated for each explanation individually and then averaged across all 200 instances.

The original AOPC is calculated using the formula in Equation 1.


(1) $$AOPC=\frac{1}{K}\sum_{i=1}^{K}\left(P(x)-P\left(x^{\left(i\right)}\right)\right)$$

where *P*(*x*) is the predicted probability for the positive class with the original input *x*, *x*^(^*^i^*^)^ is the perturbed input with the top *i* important features removed or masked, and *K* is the number of perturbation steps. This formula assumes that features contribute to the positive class; hence, their removal would result in a decrease in the predicted probability, and *P*(*x*)*–P*(*x*^(^*^i^*^)^) would be positive. Crucially, because AOPC relies on iteratively masking the top-k features, it is fundamentally a rank-based metric; it evaluates the explainer’s ability to correctly order feature importance rather than its precision in quantifying absolute attribution values.

For binary text classification, feature attributions could be associated with a negative value, indicating more support for the negative class [34]. Under such circumstances, their removal would lead to an increase in the probability of the positive class. For this reason, we adapted the AOPC formula in Equation 2.


(2) $$AOPC=\frac{1}{K_{p}+K_{n}}\left(\sum_{i=1}^{K_{p}}\left(P(x)-P\left(x^{\left(i\right)}\right)\right)+\sum_{j=1}^{K_{n}}\left(P\left(x^{\left(j\right)}\right)-P(x)\right)\right)$$

where *x*^(^*^i^*^)^ and *x*^(^*^j^*^)^ are the perturbed inputs with the top *i* positively-attributed features and the top *j* negatively-attributed features masked, respectively. *K_p_* and *K_n_* are the number of perturbation steps for the positive features and negative features, respectively, which, in this case, would be equal to the number of positively and negatively attributed tokens. Similar to the original metric, a larger value would indicate higher attribution faithfulness. Note that the operands corresponding to the “+” operation must be computed separately (to enable the removal of positive features and negative features separately) before the final summation is performed.

#### Correlation Analysis

The pairwise correlation between feature attributions for each of the 4 methods (SHAP, IG, GPT-index, and GPT-token) was assessed using Pearson *r*, Spearman *ρ*, and Kendall *τ*. Distribution similarity was measured using the Wasserstein distance. A *P* value of .05 or less is indicative of statistical significance. The distributions of feature attributions were visualized using scatter plots.

#### Feature Importance Attributions

The 10 most important features that had an occurrence of ≥1, ≥10, and ≥100 for each explainer were examined using bar graphs.

### Sensitivity Analysis

We conducted sensitivity analyses, including instances that were correctly classified only, to explore the impact of classification accuracy on explanation faithfulness.

### Hardware and Software

We used the resources from the Cedar cluster of the Digital Research Alliance of Canada. Training, evaluation, and explanation were conducted using 1 NVIDIA V100 Volta (32 GB HBM2 memory), as well as an allocation of 8 cores and 40 GB of memory. Querying of GPT was conducted locally with an AMD 9950x and 64GB system memory.

Visual Studio Code (Microsoft Corp) and Python 3.11.9 (Python Software Foundation) were used for all software development. We used the *transformers* library by Hugging Face to obtain pretrained models, and *torch* was used for evaluation purposes. The *shap* and *captum* libraries were used to calculate feature attributions via partition explainer and IG, respectively. The *openai* library was used to query GPT-4o. Data management and statistical analysis were conducted using *pandas*, *numpy*, and *scikit-learn*. Data visualization was done with *matplotlib* and *seaborn*. The full list of libraries used on the Digital Research Alliance of Canada and the local environment can be found in Table S4 in Multimedia Appendix 1.

### Ethical Considerations

This study exclusively involved the computational analysis of previously published biomedical and clinical literature originating from the McMaster PLUS and Clinical Hedges databases. As the research relied entirely on the secondary analysis of publicly available, published documents and did not involve the collection of data from or interaction with human subjects, it is exempt from institutional ethics review. Consequently, requirements regarding informed consent, human subject privacy and confidentiality protections, and participant compensation are not applicable to this study.

## Results

### Characteristics of the Dataset and Classifier

The original dataset contained 60,802 instances, of which 34,090 (56.1%) were rigorous. After stratified sampling, the 200 instances contained 83 (41.5%) rigorous studies. Within this dataset, the BioLinkBERT model achieved a cross-entropy loss of 0.527, an area under the receiver-operating characteristic curve of 0.812, and an accuracy of 0.705 using the default threshold of a predicted probability of 0.50 or more. The 200 instances contained a total of 80,901 tokens, of which 6369 were unique.

### Importance Definitions by GPT

GPT, in both prompting schemes, was instructed to define “importance” after being provided with the initial user prompt and subsequently redefine “importance” after all iterations of masking had been completed. Both GPT-index and GPT-token initially defined “importance” as the change in the predicted probability of the positive class before and after masking for all 200 instances.

After redefinition for GPT-index, the definition remained consistent as the change in predicted probability in 199 (99.5%) instances. Of these, 3 (1.5%), 37 (18.5%), and 16 (8%) instances normalized the change by logits, initial predicted probability, and the number of masked tokens in a perturbation, respectively. The remaining instance used the change in the difference between the positive and negative logits as the definition of importance.

For GPT-token, the definition for all 200 instances remained consistent as the change in predicted probability. Among these, 67 (33.5%) and 9 (4.5%) instances normalized the change by the initial predicted probability and the number of tokens masked, respectively.

### AOPC Analysis

**Figure 2.**
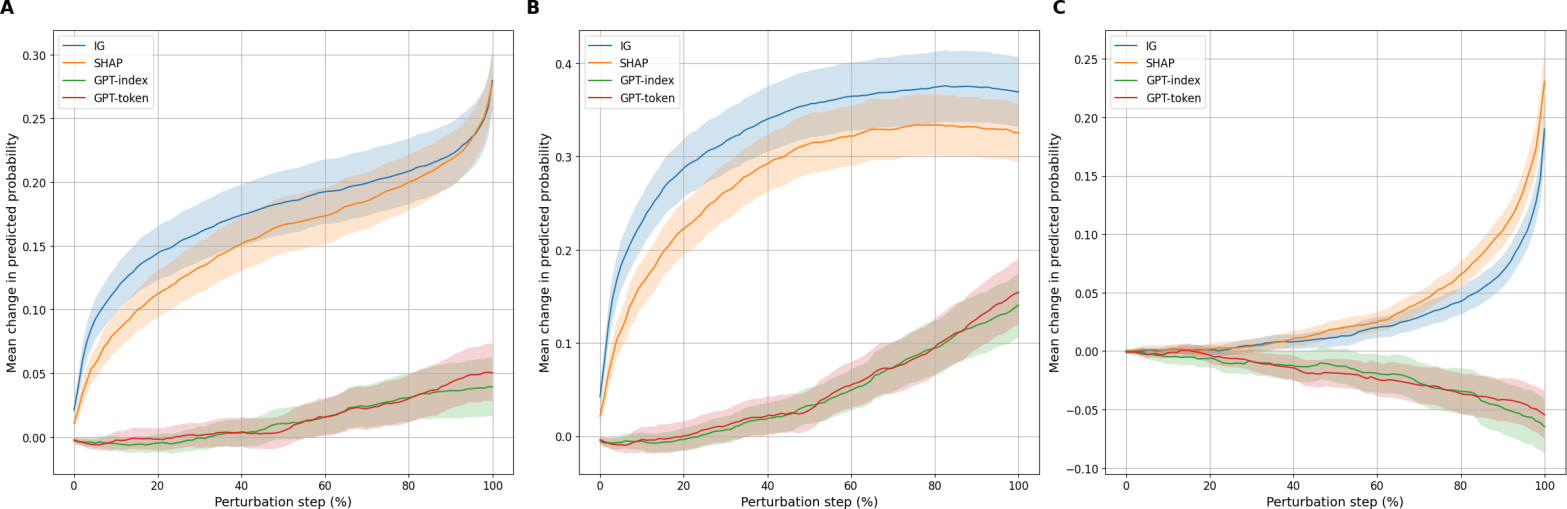
Perturbation curves of the 4 explainers (Shapley Additive Explanations [SHAP], integrated gradients [IG], GPT-index, and GPT-token) across 200 stratified studies sampled from the McMaster Premium Literature Service (PLUS) and Clinical Hedges databases (2003-2024), classified for methodological rigor using a fine-tuned BioLinkBERT model. Shaded areas represent the 95% CI. (A) All tokens, (B) tokens with positive attributions, and (C) tokens with negative attributions.

SHAP and IG explanations achieved similar faithfulness, with a mean (95% CI) of 0.222 (0.200-0.244) and 0.225 (0.202-0.247), respectively (Table 1 and Figure 2). SHAP was better at identifying negative tokens, while IG was better at identifying positive tokens. The GPT-index and GPT-token schemes yielded substantially lower AOPC scores of 0.025 (0.012-0.038) and 0.029 (0.014-0.043), respectively. Notably, both schemes produced inverted (negative) AOPC values for negative tokens, indicating a divergence in baseline attribution logic.

**Table 1.** Performance of 4 explainers (Shapley Additive Explanations [SHAP], integrated gradients [IG], GPT-index, and GPT-token) based on the mean area over the perturbation curve (AOPC) across 200 stratified studies sampled from the McMaster Premium Literature Service (PLUS) and Clinical Hedges databases (2003-2024), classified for methodological rigor using a fine-tuned BioLinkBERT model. Higher AOPC indicates greater attribution faithfulness^a^.

Explainer	AOPC (all tokens), mean (95% CI)	AOPC (Tokens with positive attributions), mean (95% CI)	AOPC (Tokens with negative attributions), mean (95% CI)
SHAP	0.222 (0.200 to 0.244)	0.277 (0.249 to 0.306)	0.037 (0.030 to 0.044)
IG	0.225 (0.202 to 0.247)	0.326 (0.293 to 0.359)	0.026 (0.019 to 0.033)
GPT-index	0.025 (0.012 to 0.038)	0.045 (0.028 to 0.063)	−0.021 (−0.034 to −0.008)
GPT-token	0.029 (0.014 to 0.043)	0.049 (0.029 to 0.068)	−0.021 (−0.031 to −0.010)

a All values are shown as the mean (95% CI) across the 200 instances.

### Sign Inversion Error Analysis

To analyze whether the negative AOPC values for negatively attributed features from the GPT explainers were a result of a systematic sign error, we systematically inverted the signs of all feature attributions and recalculated their AOPC values. After inversion, the AOPC for all, positively attributed, and negatively attributed tokens were −0.019 (−0.032 to −0.006), 0.032 (0.019-0.046), and −0.046 (−0.063 to −0.028) for GPT-index, and −0.028 (−0.043 to −0.014), 0.022 (0.011-0.034), and −0.050 (−0.070 to −0.030) for GPT-token.

### Correlation Analysis

**Figure 3.**
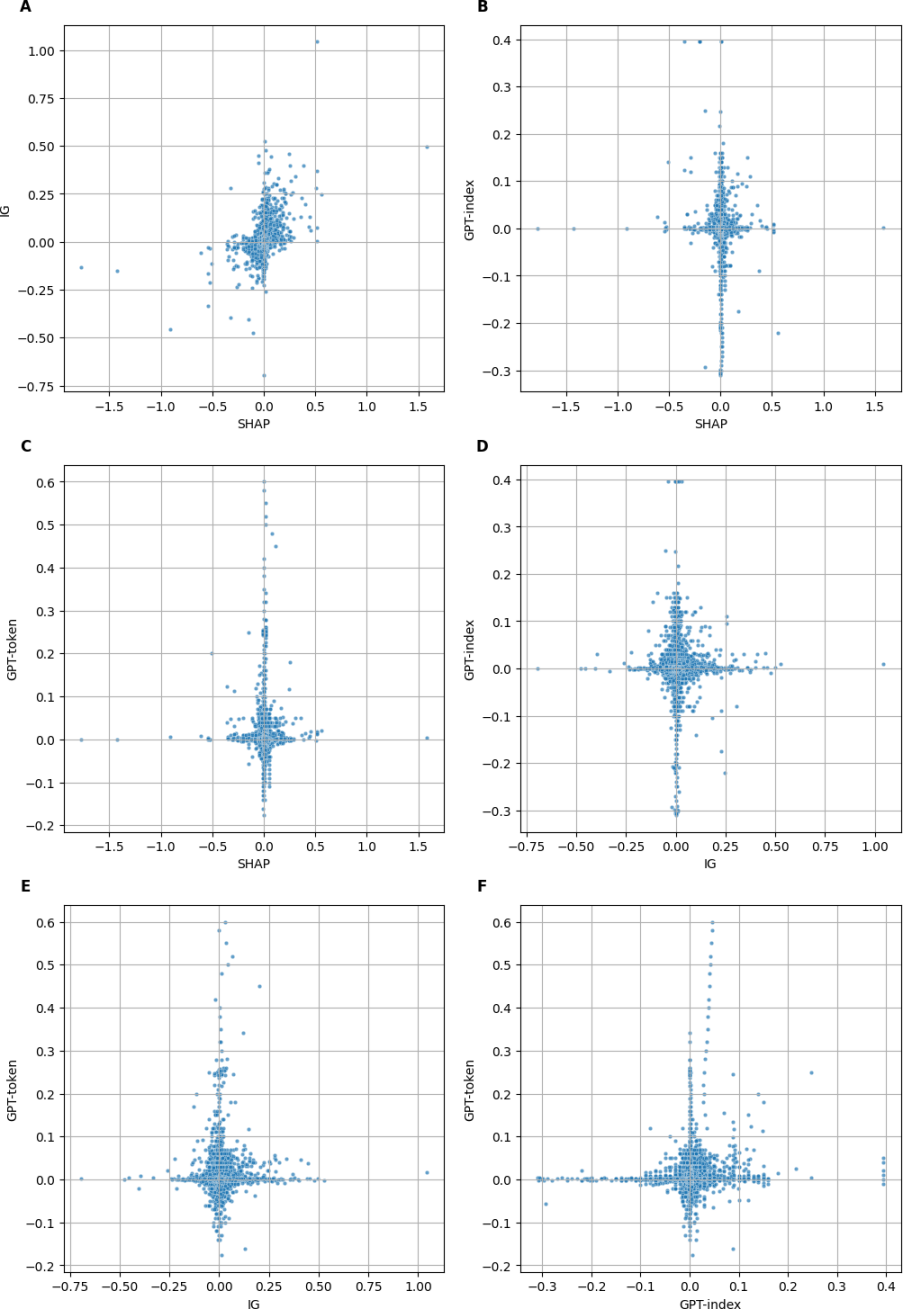
Scatter plots of token-level feature attributions generated by 4 explainers (Shapley Additive Explanations [SHAP] partition explainer, integrated gradients [IG], GPT-index, and GPT-token) across 80,901 tokens from 200 stratified studies sampled from the McMaster Premium Literature Service (PLUS) and Clinical Hedges databases (2003-2024), classified for methodological rigor using a fine-tuned BioLinkBERT model. (A) SHAP and IG, (B) SHAP and GPT-index, (C) SHAP and GPT-token, (D) IG and GPT-index, (E) IG and GPT-token, (F) GPT-index and GPT-token.

Feature attributions from SHAP and IG exhibit moderate correlation with each other, with a Pearson *r* of 0.367 (Table 2 and Figure 3). No notable correlation is evident between feature attributions from other pairs of explainers. Wasserstein distances reveal that the distributions of feature attributions are similar across all explainers.

**Table 2.** Pairwise correlation and distribution similarity of token-level feature attributions generated by 4 explainers (Shapley Additive Explanations [SHAP] partition explainer, integrated gradients [IG], GPT-index, and GPT-token) across 80,901 tokens from 200 stratified studies sampled from the McMaster Premium Literature Service (PLUS) and Clinical Hedges databases (2003-2024), classified for methodological rigor using a fine-tuned BioLinkBERT model. Pearson *r*, Spearman *ρ*, and Kendall *τ* assess linear and rank-based correlation, while the Wasserstein distance measures distributional similarity between attribution value distributions.

Explainer A	Explainer B	Pearson *r*	Spearman *ρ*	Kendall *τ*	Wasserstein distance
SHAP	IG	0.367^a^	0.275^a^	0.192^a^	0.002
SHAP	GPT-index	−0.031^a^	0.061^a^	0.041^a^	0.003
SHAP	GPT-token	0.004	0.037^a^	0.025^a^	0.003
IG	GPT-index	0.003	0.038^a^	0.026^a^	0.004
IG	GPT-token	0.032^a^	0.029^a^	0.020^a^	0.005
GPT-index	GPT-token	0.083^a^	0.096^a^	0.071^a^	0.001

a Statistical significance (*P*<.05).

### Feature Importance Attributions

**Figure 4.**
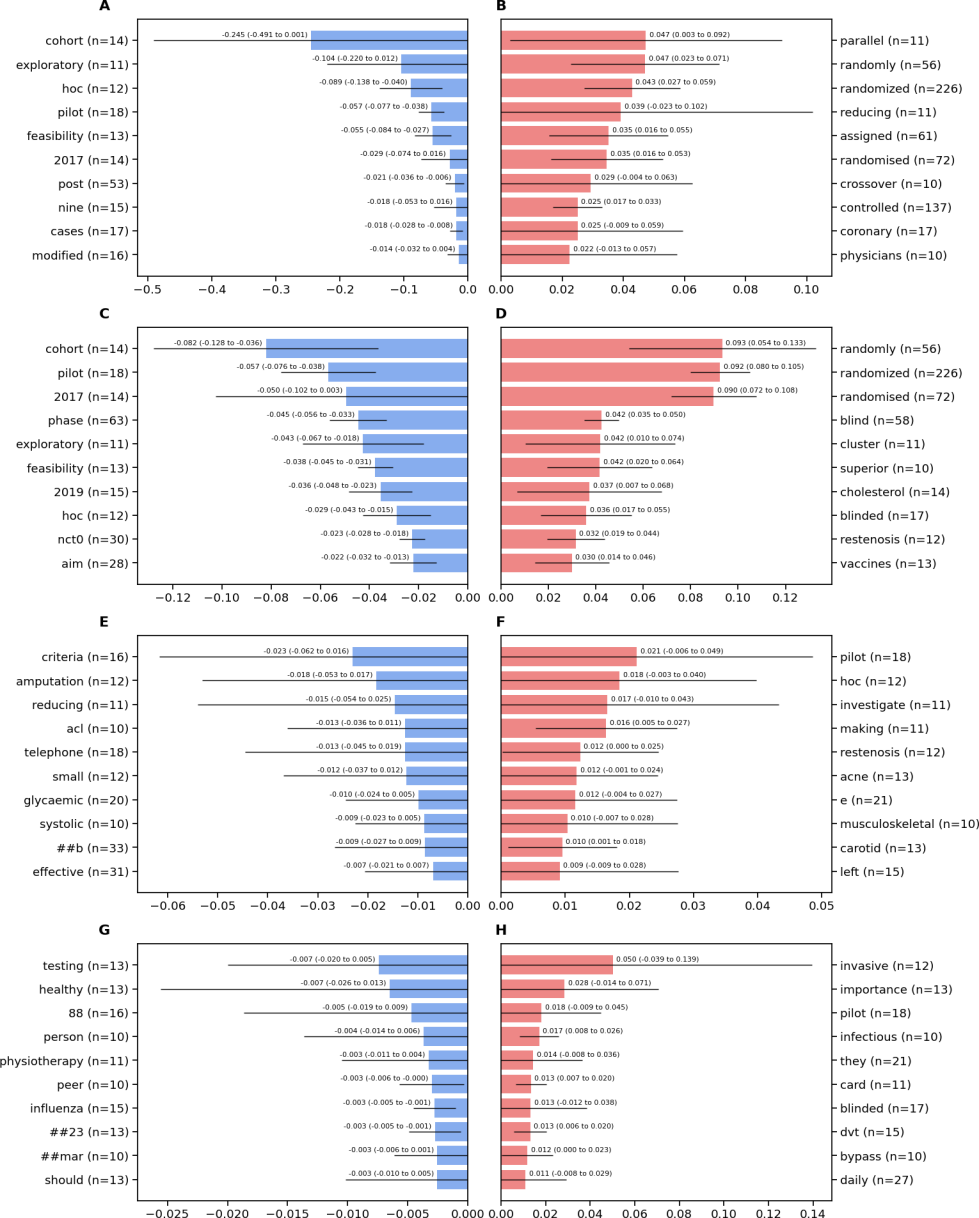
Accumulated local feature attributions of the identified most important negative and positive tokens with 10 or more occurrences, generated by 4 explainers (Shapley Additive Explanations [SHAP] partition explainer, integrated gradients [IG], GPT-index, and GPT-token) across 80,901 tokens from 200 stratified studies sampled from the McMaster Premium Literature Service (PLUS) and Clinical Hedges databases (2003 to 2024). The values are presented as mean (95% CI). (A) Negative tokens for SHAP, (B) positive tokens for SHAP, (C) negative tokens for IG, (D) positive tokens for IG, (E) negative tokens for GPT-index, (F) positive tokens for GPT-index, (G) negative tokens for GPT-token, and (H) positive tokens for GPT-token.

Of the 80,901 generated feature attributions, 6369, 1073, and 87 were from unique tokens that had occurrences of ≥1, ≥10, and ≥100, respectively. The most important unique tokens with ≥10 occurrences can be found in Figure 4. Those with occurrences of ≥1 and ≥100 can be found in Figures S1 and S2 in Multimedia Appendix 1.

Among those with ≥10 and ≥100 occurrences, both SHAP and IG identified tokens that were associated with study designs, including “cohort,” “pilot,” “exploratory,” “randomly,” and “blind,” among others. In contrast, the GPT explainers did not exhibit a cohesive semantic pattern among tokens with ≥10 occurrences. While GPT-token successfully identified select key clinical terms (eg, “trial” and “randomized”), it was unable to systematically isolate negatively contributing tokens.

Important tokens with 1 or more occurrence for SHAP and IG primarily consisted of terms related to study design, year, or topic. There is no consistent pattern between the 2 GPT explainers.

### Sensitivity Analysis

We conducted a sensitivity analysis, including only the correctly classified instances (70.5% accuracy and 141 studies). Of the 57,195 tokens, 5341, 816, and 57 were unique with an occurrence of ≥1, ≥10, and ≥100, respectively. There was no notable change in the faithfulness of the explainers based on AOPC (Table S5 and Figure S3 in Multimedia Appendix 1), pairwise correlation of feature attributions among the explainers (Table S6 and Figure S4 in Multimedia Appendix 1), and the most important tokens identified by accumulated feature attributions (Figures S5-S7 in Multimedia Appendix 1).

## Discussion

To our knowledge, this is the first experiment that attempts to leverage decoder transformers to establish feature attributions for text classifiers through perturbation. While our results do not indicate that GPT could be a potential substitute for conventional explanation methods in this context, this study nevertheless serves as a valuable exploratory analysis that could inspire future research in this area.

### Principal Findings

While AOPC does not establish absolute faithfulness, it is a common method to compare the relative performance of explainers on the same model [36]. Our results demonstrate that the SHAP partition explainer and IG were similar in their overall performance. SHAP was better at identifying negative tokens, while IG was better at identifying positive tokens. Our results also demonstrated that GPT was able to generate reasonable definitions of importance when provided with the task of generating feature attributions as an explainer. While delegating the definition of feature importance to the model itself theoretically risks inconsistency and could weaken methodological rigor, our analysis showed stability, with the model consistently defining importance as the change in predicted probability. This confirms that the poor performance of the GPT explainers is not a byproduct of an unstable metric definition. In spite of this, the GPT explainers struggled to generate reliable feature attributions. In particular, the negative AOPC for negative tokens indicates that the GPT explainers mistakenly associated negative attributions with features that increased the probability of rigor. A plausible explanation for this is a sign inversion error, wherein the model reports the raw negative delta of a masked positive feature rather than its intended importance magnitude. However, our generative prompts explicitly enforced a strict sign convention requiring positive floats for positive classifications and negative floats for negative classifications. An examination of the most important tokens reveals that the top negative features identified by GPT do not symmetrically align with the positive features identified by SHAP or IG. After sign inversion, AOPC decreased from 0.025 to −0.019 for GPT-index, and from 0.029 to −0.028 for GPT-token. Therefore, this discrepancy likely represents a limitation in GPT’s semantic feature attribution logic rather than a sign inversion or delta reporting error. These findings were echoed by the correlation analyses, where attributions by SHAP and IG had a moderate correlation with each other, while the 2 GPT explainers had weak or no correlation with the others. Also, sensitivity analyses isolating only the correctly classified instances yielded similar trends, confirming that GPT’s poor attribution performance is an inherent limitation of its logical reasoning rather than an artifact of attempting to explain confused or incorrect model predictions.

GPT’s limited faithfulness is unlikely to be attributable to an undersampled perturbation space. The initial masking generated the same number of perturbations as the number of tokens. Subsequently, GPT proceeded with 10 iterations of masking, generating between 10 and 30 masking combinations per iteration, evaluating an additional 100 to 300 unique perturbations. In contrast, the SHAP partition explainer successfully established faithful baseline attributions using only 86 perturbations for a 512-token sequence. The fact that GPT evaluated a significantly larger subset of the perturbation space yet failed to produce aligned attributions indicates an inherent limitation in the LLM’s ability to logically synthesize mathematical perturbation results, rather than a lack of search space exploration. Furthermore, it is important to emphasize that our results are established with a stratified sample of 200 instances and a specific prompting strategy. Therefore, our findings should be framed as a specific evaluation of this methodology on this dataset, rather than a definitive ruling on GPT’s overall use for all biomedical text explanations.

While methods to examine the global attributions for transformer models are an area of active research [37], we were able to examine the accumulated local attributions across all 200 instances. SHAP and IG indicate that the BioLinkBERT model generally aligned with the manual appraisal criteria [38], with terms such as “cohort,” “pilot,” “randomized,” and “blind,” among others, being identified as the most important. The tokens identified by GPT did not align with SHAP or IG and seemed to be semantically nonsensical in the context of rigor classification. For instance, both GPT-index and GPT-token identified “pilot” as a positive contributor, contrary to manual appraisal as well as SHAP and IG explanations.

### Prompting

A challenge of this experiment was the development of prompts for GPT, considering the complex nature of generating feature attributions from perturbations. It is known that sophisticated prompting techniques can improve GPT’s performance in NLP [38-40]. In our study, we used numerous established techniques in prompt engineering in an attempt to improve performance, including role prompting, decomposition by providing instructions step by step, as well as chain-of-thought to a certain degree, with multiple iterations of perturbations and the redefinition of importance [40]. GPT was also limited in responding with long, quantitative sequences despite explicit instructions and structured output restrictions [41,42]. We mitigated this concern by explicitly instructing GPT to respond with a certain number of lists as parameters to the *mask_and_predict* function, using structured outputs and function calling, and decomposing the calculation steps to 20 tokens per batch. Despite this, GPT was not able to generate faithful attributions. Furthermore, we hypothesized that an advantage of LLMs would be the ability to recognize likely important tokens before any quantitative explanations have been generated, considering their ability to understand and encode contextualized information from plain text [43]. Therefore, we experimented with 2 prompting schemes, namely GPT-index and GPT-token. However, our results show that there was no meaningful difference regardless of the inclusion of input tokens in the initial user prompt.

### Resource Requirements

A challenge with traditional XAI methods is the significant computational resources required. As previously mentioned, the exhaustive nature of calculating SHAP values from all possible perturbations is infeasible, resulting in the rise of numerous methods to approximate SHAP values [44,45], including the partition explainer [44,45]. The computational requirement for IG is associated with integration steps. While higher steps result in higher precision, we found 30 steps to be feasible on GPUs with 32GB of memory and temporally more efficient than the SHAP partition explainer.

High computational costs and time delays were incurred due to the iterative approach with the GPT explainers. Similar to SHAP, the BioLinkBERT model must be queried to obtain predictions for the perturbed instances. Additionally, each subsequent prompt in the chain results in higher inference and response times due to network latency and the autoregressive nature of LLM text generation. Consequently, GPT was unequivocally the slowest method to generate explanations, while also incurring a direct financial cost from OpenAI’s servers of approximately US $1.00 per instance.

### Deployment and Research Implications

Explainability and interpretability in biomedical and clinical machine learning are key areas of research [46,47]. As a pioneer in evidence-based medicine and knowledge translation, the McMaster Health Information Research Unit aims not only to automate biomedical literature classification and appraisal [25,48] but also to ensure that the process is transparent and reproducible to facilitate trust among clinicians who subscribe to PLUS and PLUS-associated services. Based on the results of this experiment, we believe that both SHAP and IG would be suitable for deployment alongside a top-performing model. More recently, studies [49,50] and systematic review support systems [51-53] have begun to leverage supervised or active learning extensively to support knowledge translation and synthesis by relevance ranking or automatic classification. We believe that systems should attempt to integrate XAI frameworks alongside any black-box models for better transparency.

While we did not obtain promising results in using GPT as an end-to-end approach for feature attributions, our work nevertheless serves as a foundation for future research. Given the sensitivity of GPT-based explanations to prompt design, future studies could explore more sophisticated, domain-tailored prompting strategies and iterative prompt refinement using techniques, such as few-shot learning, to better align GPT’s output with domain-specific interpretability criteria [40]. Fine-tuning LLM explainers on biomedical corpora could also improve their understanding of specialized terminology and context [54]. Hybrid explanation frameworks, such as leveraging LLMs to establish a partition hierarchy [16,30] or integrating model-internal signals, such as attention weights, with LLM-based explanation methods, may also be of interest [55-57]. Specifically, future proof-of-concept studies should investigate whether grounding LLM-generated contextual explanations in traditional feature attributions, such as SHAP or IG, can produce more faithful and human-interpretable results than standalone generative explainers.

### Strengths and Limitations

Our study has several strengths. First, to our knowledge, this is the first experiment that attempts to leverage decoder transformers to establish feature attributions for text classifiers by perturbation. Second, a concern with leveraging LLMs in medical research is reproducibility, as evidence-based medicine is founded upon concepts of transparency, reliability, and the ability to validate findings through rigorous, repeatable methodologies [58-61]. We mitigated this concern by using a temperature of 0, making the outputs of the LLM deterministic and replicable. Third, we mitigated concerns with the original AOPC metric on binary text classification by separately considering negative and positive features. This allowed us to better capture the faithfulness of the explanations. Fourth, we leveraged sophisticated prompting techniques for GPT. This indicates that the poor results from GPT are likely an inherent limitation of the pretraining and the model architecture rather than the prompt.

Nevertheless, important limitations must be considered when interpreting our results. First, there is no known method to establish ground truth in black-box models, and explaining text models with a high feature space remains a challenge [36,62]. Consequently, SHAP and IG were used as established comparative baselines rather than definitive ground truths, and AOPC was used as an objective proxy for faithfulness. For IG specifically, the “[PAD]” baseline yields a predicted probability of 11.7% for the positive class, compared to the class prevalence of 41.5% in the stratified subset, indicating that the baseline is not prediction-neutral. This means IG attributions reflect token contributions relative to a negatively-biased starting point, which may systematically inflate the apparent importance of tokens that are most associated with the positive class. For these reasons, it is important to note that our findings are context-specific, dataset-specific, and model-specific. Second, due to resource constraints, we could only experiment with a stratified subset of 200 instances from the original dataset. While we attempted to minimize sampling bias through stratified sampling, this limited sample size may restrict the generalizability of our findings, particularly regarding the correlation analysis. However, because the unit of analysis for feature attribution is the token (N=80,901), our study retains robust statistical power to evaluate explainer behavior within this sample, as evidenced by our narrow CI. Nonetheless, a larger dataset would further increase our confidence. Third, word-piece tokenization often separates words into fragments, potentially affecting how feature attributions are assigned [63]. This mismatch between models may have contributed to the poor performance of the GPT-token scheme, forcing a generative LLM to reason over another model’s disjointed, comma-separated word-piece tokens rather than its native text processing. Consequently, the explanations may not correspond to human-interpretable linguistic units, especially for numerical texts. However, the negligible performance difference between GPT-token and GPT-index, which was only provided the number of maskable tokens, indicates that tokenization is likely not the primary contributor to explanation faithfulness. Nevertheless, as a language model, GPT may be limited in accurately mapping tokens from a long, comma-separated list of numerical indices. Therefore, future research should investigate whether recombining these subword tokens into whole words prior to the LLM explanation phase improves semantic alignment and attribution faithfulness. Finally, GPT’s performance on a task is heavily prompt-specific. While our methodology used highly sophisticated prompt engineering techniques, our evaluation was strictly zero-shot to test the model’s baseline reasoning. The substantial API costs associated with iterative perturbation precluded us from conducting comprehensive ablation studies or providing few-shot examples. Furthermore, both SHAP and IG are of a zero-shot nature, and using zero-shot for GPT allows for a more robust comparison. It remains unknown whether GPT would show promise with a different set of prompts, and this is a critical area for future investigation.

### Conclusions

We conducted a comprehensive proof-of-concept exploration into the application of GPT-4o as a standalone, end-to-end perturbation explainer for a BioLinkBERT biomedical text classifier. Our objective was to compare the faithfulness of GPT-driven explanations against established baseline methods, specifically the SHAP partition explainer and IG. The results demonstrated that while SHAP and IG provided consistent and relatively faithful feature attributions, the GPT-based approaches, regardless of whether they were prompted with token indices or explicit subword tokens, yielded poor explanations. This was evidenced by near-zero correlation with established methods and counterintuitive token attributions. Consequently, the findings of this study indicate that despite advanced contextual capabilities, current generative LLMs struggle to accurately synthesize mathematical feature importance through iterative masking, lacking the reliability of traditional XAI frameworks for this specific task. Despite these limitations, our work offers valuable insights and establishes a foundation for future research aimed at integrating LLMs into the explainability framework.

## Supplementary material

10.2196/81644 Multimedia Appendix 1 Prompt definitions, software environments, and additional analyses.

## Abbreviations

AOPC: area over the perturbation curve
API: application programming interface
BERT: Bidirectional Encoder Representations from Transformers
IG: integrated gradients
LLM: large language model
NLP: natural language processing
PLUS: Premium Literature Service
SHAP: Shapley Additive Explanations
XAI: explainable artificial intelligence
