# Supplementary material for: Understanding Transformer-Based Classifications of Medical Text Using a Large Language Model for the Attribution of Feature Importance: Proof-of-Concept Algorithm Development and Validation Study
Source: JMIR Med Inform. 2026 Jun 10;14:e81644. doi: 10.2196/81644 (PMC13252589; doi:10.2196/81644)
Supplement: Multimedia Appendix 1 [file medinform-v14-e81644-s001.docx]

Supplementary Information

[Table S1. GPT prompt dictionary mapping prompt keys to prompt content for GPT-index and GPT-token. 1](#_Toc228274419)

[Table S2. JSON object dictionary mapping JSON keys to object type in Python for GPT API’s structured output function. 3](#_Toc228274420)

[Table S3. Chain of message keys for generating feature attributions with GPT. Assistant messages indicate replies from GPT. 4](#_Toc228274421)

[Table S4. Software environment and libraries used locally and in the cloud on the Cedar cluster (now retired) of the Digital Research Alliance of Canada. 6](#_Toc228274422)

[Table S5. Performance of four explainers (SHAP, IG, GPT-index, GPT-token) based on mean area over the perturbation curve (AOPC) across 141 correctly classified stratified articles sampled from the McMaster PLUS and Clinical Hedges databases (2003 to 2024), classified for methodological rigour using a fine-tuned BioLinkBERT model. Higher AOPC indicates greater attribution faithfulness. 10](#_Toc228274423)

[Table S6. Pairwise correlation and distribution similarity of token-level feature attributions generated by four explainers (SHAP partition explainer, integrated gradients, GPT-index, and GPT-token) across 57,195 tokens from the 141 correctly classified stratified articles sampled from the McMaster PLUS and Clinical Hedges databases (2003 to 2024), classified for methodological rigour using a fine-tuned BioLinkBERT model. Pearson's r, Spearman's ρ, and Kendall's τ assess linear and rank-based correlation; Wasserstein distance measures distributional similarity between attribution value distributions. 10](#_Toc228274424)

[Figure S1. Accumulated local feature attributions of the identified most important negative and positive tokens with ≥1 occurrences generated by four explainers (SHAP partition explainer, integrated gradients, GPT-index, and GPT-token) across 80,901 tokens from 200 stratified articles sampled from the McMaster PLUS and Clinical Hedges databases (2003 to 2024). Values are mean and 95% CI. 11](#_Toc228274425)

[Figure S2. Accumulated local feature attributions of the identified most important negative and positive tokens with ≥100 occurrences generated by four explainers (SHAP partition explainer, integrated gradients, GPT-index, and GPT-token) across 80,901 tokens from 200 stratified articles sampled from the McMaster PLUS and Clinical Hedges databases (2003 to 2024). Values are mean and 95% CI. 12](#_Toc228274426)

[Figure S3. Perturbation curves of the four explainers (SHAP, IG, GPT-index, GPT-token) across 141 correctly classified stratified articles sampled from the McMaster PLUS and Clinical Hedges databases (2003 to 2024), classified for methodological rigour using a fine-tuned BioLinkBERT model. Shaded areas represent the 95% CI. 13](#_Toc228274427)

[Figure S4. Scatter plots of token-level feature attributions generated by four explainers (SHAP partition explainer, integrated gradients, GPT-index, and GPT-token) across 57,195 tokens from 141 correctly classified stratified articles sampled from the McMaster PLUS and Clinical Hedges databases (2003 to 2024), classified for methodological rigour using a fine-tuned BioLinkBERT model. 14](#_Toc228274428)

[Figure S5. Accumulated local feature attributions of the identified most important negative and positive tokens with ≥1 occurrences generated by four explainers (SHAP partition explainer, integrated gradients, GPT-index, and GPT-token) across 57,195 tokens from 141 correctly classified stratified articles sampled from the McMaster PLUS and Clinical Hedges databases (2003 to 2024). Values are mean and 95% CI. 15](#_Toc228274429)

[Figure S6. Accumulated local feature attributions of the identified most important negative and positive tokens with ≥10 occurrences generated by four explainers (SHAP partition explainer, integrated gradients, GPT-index, and GPT-token) across 57,195 tokens from 141 correctly classified stratified articles sampled from the McMaster PLUS and Clinical Hedges databases (2003 to 2024). Values are mean and 95% CI. 16](#_Toc228274430)

# Table S1. GPT prompt dictionary mapping prompt keys to prompt content for GPT-index and GPT-token.

| **Prompt Key** | **Prompt Content** |
| --- | --- |
| ROLE | You are a machine learning model explainer. |
| TASK | You are tasked with explaining binary text classification encoder-only transformer model's predictions by perturbing its input tokens, similar to perturbation based XAI frameworks like LIME or SHAP. |
| CRITERIA | Manual appraisal criteria:  All of these criteria must be met to be rated as being rigorous. The article will be rated as not rigorous if any criteria are not met. The criteria are 1) in English, 2) about humans, 3) about topics that are important to the clinical practice of medicine, nursing, rehabilitation, and other health professions, other than descriptive studies of prevalence, 4) analysis of each article consistent with the study question, 5) random allocation of participants to comparison groups, 6) 10 or more patients per group completing primary outcome assessment, 7) primary outcome(s) assessed in 80% or more of those randomized at the defined follow-up point, 8) primary outcome is clinically important or ≥1 secondary outcome is clinically important, and 9) subgroup analyses must be preplanned, with groups analyzed as they were randomized; analyses must test for interaction between 2 or more subgroups. |
| PROVIDED_INFO_INDEX | You will be provided the number of tokens, the logits for both positive and negative classes, and the probability for the positive class. You will NOT be provided the text. |
| INSTRUCTIONS_INDEX | For a given instance, determine which tokens have the greatest impact on the model's prediction by systematically masking them. You will:  1. Receive an instance with the number of tokens and model outputs without any masking.  2. Define 'importance' for yourself. The importance should be a float. A negative and positive float should indicate that the token increases the chance of a negative and positive classification, respectively.  3. Generate a list of num_token number of lists where each list contains the index of 1 token to mask (e.g., [[0], [1], [2], [3], ...]). Tokens corresponding to the indexes will be replaced by `[MASK]` by the `mask_and_predict` function.  4. Repeat steps 4a and 4b for 10 iterations. Once all 10 iterations are complete, you will be prompted to proceed to step 5.  4a. Generate a list of lists of one or numerous index(es) to mask, based on the results of previous iterations. Start with completely random number of lists and indexes and adjust the number of lists and indexes in each list based on model outputs. DO NOT repeatedly mask the same combination of tokens.  4b. Call `mask_and_predict` with the lists of indexes to mask. The function will return a list of lists in the form of [logit_positive, logit_negative, probability_positive], in which each list corresponds with the model's output given the masked variant.  5. You will make any adjustments to your initial definition of 'importance'.  6. You will be prompted with indexes of the tokens. Please calculate the importance of each prompted token in the text.  7. For each token, output the index and the importance. |
| DEVELOPER_INDEX | {ROLE} {TASK}  {PROVIDED_INFO_INDEX}  {INSTRUCTIONS_INDEX} |
| INITIAL_USER_INDEX | Number of tokens: <num_tokens>.  Model output without masking: [[<logit_positive>, <logit_negative>, <probability_positive>]]. |
| PROVIDED_INFO_TOKEN | You will be provided the number of tokens, the input tokens, the logits for both positive and negative classes, the probability for the positive class. You will also be provided the manual appraisal criteria (appraised using the full text). |
| INSTRUCTIONS_TOKEN | For a given instance, determine which tokens have the greatest impact on the model's prediction by systematically masking them. You will:  1. Receive an instance with the number of tokens, the tokens, and model outputs without any masking.  2. Define 'importance' for yourself. The importance should be a float. A negative and positive float should indicate that the token increases the chance of a negative and positive classification, respectively.  3. Generate a list of num_token number of lists where each list contains the index of 1 token to mask (e.g., [[0], [1], [2], [3], ...]). Tokens corresponding to the indexes will be replaced by `[MASK]` by the `mask_and_predict` function.  4. Repeat steps 4a and 4b for 10 iterations. Once all 10 iterations are complete, you will be prompted to proceed to step 5.  4a. Generate a list of lists of one or numerous index(es) to mask, based on which token you think is semantically important and the results of previous masking iterations. DO NOT repeatedly mask the same combination of tokens.  4b. Call `mask_and_predict` with the lists of indexes to mask. The function will return a list of lists in the form of [logit_positive, logit_negative, probability_positive], in which each list corresponds with the model's output given the masked variant.  5. You will make any adjustments to your initial definition of 'importance'.  6. You will be prompted with indexes of the tokens. Please calculate the importance of each prompted token in the text.  7. For each token, output the index and the importance. |
| DEVELOPER_TOKEN | {ROLE} {TASK}  {PROVIDED_INFO_TOKEN}  {INSTRUCTIONS_TOKEN}  {CRITERIA} |
| INITIAL_USER_TOKEN | Number of tokens: <num_tokens>.  Input tokens: <token_list>  Model output without masking: [[<logit_positive>, <logit_negative>, <probability_positive>]]. |
| DEFINE_IMPORTANCE | Please define 'importance'. You will use this definition to calculate token importance when prompted later. |
| INDIVIDUAL_MASKING | Please generate the initial masking list and call 'mask_and_predict'. The input must be a list of <num_tokens> lists of one index each. |
| ITERATION_MASKING | Please proceed with iteration <iteration> of masking. Generate 10 to 30 lists. Only generate indexes from 0 to <num_tokens – 1>. Add, remove, or adjust masking based on previous maskings and model outputs. Prioritize masking tokens that seemed to be important from previous iterations AND tokens that have rarely been masked in previous iterations. DO NOT repeatedly mask the same combination of tokens. DO NOT reply with anything else other than the function call. |
| REDEFINE_IMPORTANCE | Based on the results of all previous masking iterations, adjust the definition of 'importance' for yourself as you see fit. |
| ATTRIBUTION_CALCULATION | Please calculate token importance for tokens from index <i> to <minimum(i + 20 - 1, num_token - 1)> based on previous information and your definition of 'importance'. |
| MASK_AND_PREDICT_OUTPUTS | [[<logit_positive_0>, <logit_negative_0>, <probability_positive_0>] …] |

# Table S2. JSON object dictionary mapping JSON keys to object type in Python for GPT API’s structured output function.

| **Object Key** | **Object Type** |
| --- | --- |
| TOKEN_INDEX | int |
| IMPORTANCE_VALUE | float |
| IMPORTANCE_DEFINITION | str |
| TOKEN_IMPORTANCE | TOKEN_INDEX: IMPORTANCE_VALUE |
| TOKEN_IMPORTANCE_RESULTS | [TOKEN_IMPORTANCE] |
| MASK_LIST | [int] |
| MASK_LISTS | [MASK_LIST] |

# Table S3. Chain of message keys for generating feature attributions with GPT. Assistant messages indicate replies from GPT.

| **Sequence** | **Role** | **Key** |
| --- | --- | --- |
| 1 | developer | DEVELOPER_INDEX/DEVELOPER_TOKEN |
| 2 | user | INITIAL_USER_INDEX/INITIAL_USER_TOKEN |
| 3 | user | DEFINE_IMPORTANCE |
| 4 | assistant | IMPORTANCE_DEFINITION |
| 5 | user | INDIVIDUAL_MASKING |
| 6 | assistant | MASK_LISTS |
| 7 | tool | MASK_AND_PREDICT_OUTPUTS |
| 8 | user | ITERATION_MASKING |
| 9 | assistant | MASK_LISTS |
| 10 | tool | MASK_AND_PREDICT_OUTPUTS |
| 11 | user | ITERATION_MASKING |
| 12 | assistant | MASK_LISTS |
| 13 | tool | MASK_AND_PREDICT_OUTPUTS |
| 14 | user | ITERATION_MASKING |
| 15 | assistant | MASK_LISTS |
| 16 | tool | MASK_AND_PREDICT_OUTPUTS |
| 17 | user | ITERATION_MASKING |
| 18 | assistant | MASK_LISTS |
| 19 | tool | MASK_AND_PREDICT_OUTPUTS |
| 20 | user | ITERATION_MASKING |
| 21 | assistant | MASK_LISTS |
| 22 | tool | MASK_AND_PREDICT_OUTPUTS |
| 23 | user | ITERATION_MASKING |
| 24 | assistant | MASK_LISTS |
| 25 | tool | MASK_AND_PREDICT_OUTPUTS |
| 26 | user | ITERATION_MASKING |
| 27 | assistant | MASK_LISTS |
| 28 | tool | MASK_AND_PREDICT_OUTPUTS |
| 29 | user | ITERATION_MASKING |
| 30 | assistant | MASK_LISTS |
| 31 | tool | MASK_AND_PREDICT_OUTPUTS |
| 32 | user | ITERATION_MASKING |
| 33 | assistant | MASK_LISTS |
| 34 | tool | MASK_AND_PREDICT_OUTPUTS |
| 35 | user | ITERATION_MASKING |
| 36 | assistant | MASK_LISTS |
| 37 | tool | MASK_AND_PREDICT_OUTPUTS |
| 38 | user | REDEFINE_IMPORTANCE |
| 39 | assistant | IMPORTANCE_DEFINITION |
| 40 | user | ATTRIBUTION_CALCULATION |
| 41 | assistant | TOKEN_IMPORTANCE_RESULTS |

# Table S4. Software environment and libraries used locally and in the cloud on the Cedar cluster (now retired) of the Digital Research Alliance of Canada.

| **Library** | **Local Version** | **Cloud Version** |
| --- | --- | --- |
| #paycheck | 1.0.2 | N/A |
| #torch | 2.2.1 | N/A |
| Cython | 0.29.36 | 0.29.36+computecanada |
| GitPython | 3.1.40 | 3.1.40+computecanada |
| Jinja2 | 3.1.2 | 3.1.2+computecanada |
| MarkupSafe | 2.1.3 | 2.1.3+computecanada |
| Pillow | 10.0.0 | 10.0.0+computecanada |
| PyNaCl | 1.5.0 | 1.5.0+computecanada |
| PyYAML | 6.0.1 | 6.0.1+computecanada |
| Pygments | 2.16.1 | 2.16.1+computecanada |
| Send2Trash | 1.8.2 | 1.8.2+computecanada |
| accelerate | 0.27.2 | 0.27.2+computecanada |
| aiohttp | 3.9.1 | 3.9.1+computecanada |
| aiosignal | 1.3.1 | 1.3.1+computecanada |
| anyio | 3.7.1 | 3.7.1+computecanada |
| appdirs | 1.4.4 | 1.4.4+computecanada |
| arff | 0.9 | 0.9+computecanada |
| argon2_cffi | 23.1.0 | 23.1.0+computecanada |
| argon2_cffi_bindings | 21.2.0 | 21.2.0+computecanada |
| asttokens | 2.2.1 | 2.2.1+computecanada |
| async_generator | 1.1 | 1.10+computecanada |
| attrs | 23.1.0 | 23.1.0+computecanada |
| backcall | 0.2.0 | 0.2.0+computecanada |
| backports-abc | 0.5 | 0.5+computecanada |
| backports.shutil_get_terminal_size | 1.0.0 | 1.0.0+computecanada |
| bcrypt | 4.0.1 | 4.0.1+computecanada |
| beautifulsoup4 | 4.12.2 | 4.12.2+computecanada |
| bitarray | 2.8.1 | 2.8.1+computecanada |
| bitstring | 4.1.1 | 4.1.1+computecanada |
| bleach | 6.0.0 | 6.0.0+computecanada |
| captum | N/A | 0.3.0+computecanada |
| certifi | 2023.7.22 | 2023.7.22+computecanada |
| cffi | 1.15.1 | 1.15.1+computecanada |
| chardet | 5.2.0 | 5.2.0+computecanada |
| charset_normalizer | 3.2.0 | 3.2.0+computecanada |
| click | 8.1.7 | 8.1.7+computecanada |
| comm | 0.1.4 | 0.1.4+computecanada |
| contourpy | 1.1.0 | 1.1.0+computecanada |
| cryptography | 39.0.1 | 39.0.1+computecanada |
| cycler | 0.11.0 | 0.11.0+computecanada |
| datasets | 2.18.0 | 2.18.0+computecanada |
| deap | 1.4.1 | 1.4.1+computecanada |
| debugpy | 1.6.7.post1 | 1.6.7.post1+computecanada |
| decorator | 5.1.1 | 5.1.1+computecanada |
| defusedxml | 0.7.1 | 0.7.1+computecanada |
| dill | 0.3.8 | 0.3.8+computecanada |
| dnspython | 2.4.2 | 2.4.2+computecanada |
| docker-pycreds | 0.4.0 | 0.4.0+computecanada |
| ecdsa | 0.18.0 | 0.18.0+computecanada |
| entrypoints | 0.4 | 0.4+computecanada |
| evaluate | 0.4.1 | 0.4.2+computecanada |
| executing | 1.2.0 | 1.2.0+computecanada |
| fastjsonschema | 2.18.0 | 2.18.0+computecanada |
| filelock | 3.13.1 | 3.13.1+computecanada |
| fonttools | 4.42.1 | 4.42.1+computecanada |
| frozenlist | 1.4.1 | 1.4.1+computecanada |
| fsspec | 2024.2.0 | 2024.2.0+computecanada |
| funcsigs | 1.0.2 | 1.0.2+computecanada |
| gitdb | 4.0.11 | 4.0.11+computecanada |
| huggingface_hub | 0.21.4 | 0.21.4+computecanada |
| idna | 3.4 | 3.4+computecanada |
| importlib_metadata | 6.8.0 | 6.8.0+computecanada |
| importlib_resources | 6.0.1 | 6.0.1+computecanada |
| ipykernel | 6.25.1 | 6.25.1+computecanada |
| ipython | 8.15.0 | 8.15.0+computecanada |
| ipython_genutils | 0.2.0 | 0.2.0+computecanada |
| jedi | 0.19.0 | 0.19.0+computecanada |
| joblib | 1.3.2 | 1.3.2+computecanada |
| jsonschema | 4.19.0 | 4.19.0+computecanada |
| jsonschema_specifications | 2023.7.1 | 2023.7.1+computecanada |
| jupyter_client | 8.3.1 | 8.3.1+computecanada |
| jupyter_core | 5.3.1 | 5.3.1+computecanada |
| kiwisolver | 1.4.5 | 1.4.5+computecanada |
| lockfile | 0.12.2 | 0.12.2+computecanada |
| matplotlib | 3.7.2 | 3.7.2+computecanada |
| matplotlib_inline | 0.1.6 | 0.1.6+computecanada |
| mistune | 3.0.1 | 3.0.1+computecanada |
| mock | 5.1.0 | 5.1.0+computecanada |
| mpmath | 1.3.0 | 1.3.0+computecanada |
| multidict | 6.0.5 | 6.0.5+computecanada |
| multiprocess | 0.70.16 | 0.70.16+computecanada |
| nest_asyncio | 1.5.7 | 1.5.7+computecanada |
| netaddr | 0.8.0 | 0.8.0+computecanada |
| netifaces | 0.11.0 | 0.11.0+computecanada |
| networkx | 3.2.1 | 3.2.1+computecanada |
| nose | 1.3.7 | 1.3.7+computecanada |
| numpy | 1.25.2 | 1.25.2+computecanada |
| packaging | 23.1 | 23.1+computecanada |
| pandas | 2.1.0 | 2.1.0+computecanada |
| pandocfilters | 1.5.0 | 1.5.0+computecanada |
| paramiko | 3.3.1 | 3.3.1+computecanada |
| parso | 0.8.3 | 0.8.3+computecanada |
| path | 16.7.1 | 16.7.1+computecanada |
| path.py | 12.5.0 | 12.5.0+computecanada |
| pathlib2 | 2.3.7.post1 | 2.3.7.post1+computecanada |
| paycheck | N/A | 1.0.2+computecanada |
| pbr | 5.11.1 | 5.11.1+computecanada |
| pexpect | 4.8.0 | 4.8.0+computecanada |
| pickleshare | 0.7.5 | 0.7.5+computecanada |
| pkgutil_resolve_name | 1.3.10 | 1.3.10+computecanada |
| platformdirs | 3.9.1 | 3.9.1+computecanada |
| prometheus_client | 0.17.1 | 0.17.1+computecanada |
| prompt_toolkit | 3.0.39 | 3.0.39+computecanada |
| protobuf | 4.25.2 | 4.25.2+computecanada |
| psutil | 5.9.5 | 5.9.5+computecanada |
| ptyprocess | 0.7.0 | 0.7.0+computecanada |
| pure_eval | 0.2.2 | 0.2.2+computecanada |
| pyarrow | 15.0.1 | 15.0.1 |
| pyarrow_hotfix | 0.6 | 0.6+computecanada |
| pycparser | 2.21 | 2.21+computecanada |
| pyparsing | 3.0.9 | 3.0.9+computecanada |
| pyrsistent | 0.19.3 | 0.19.3+computecanada |
| python-dateutil | 2.8.2 | 2.8.2+computecanada |
| python_json_logger | 2.0.7 | 2.0.7+computecanada |
| pytz | 2023.3 | 2023.3+computecanada |
| pyzmq | 25.1.1 | 25.1.1+computecanada |
| referencing | 0.30.2 | 0.30.2+computecanada |
| regex | 2023.8.8 | 2023.8.8+computecanada |
| requests | 2.31.0 | 2.31.0+computecanada |
| responses | 0.18.0 | 0.18.0+computecanada |
| rfc3339_validator | 0.1.4 | 0.1.4+computecanada |
| rfc3986_validator | 0.1.1 | 0.1.1+computecanada |
| rpds_py | 0.10.0 | 0.10.0+computecanada |
| safetensors | 0.4.1 | 0.4.1+computecanada |
| scikit_learn | 1.3.1 | 1.3.1+computecanada |
| scipy | 1.11.2 | 1.11.2+computecanada |
| sentry_sdk | 1.38.0 | 1.38.0+computecanada |
| setproctitle | 1.3.2 | 1.3.2+computecanada |
| shap | N/A | 0.43.0+computecanada |
| simplegeneric | 0.8.1 | 0.8.1+computecanada |
| singledispatch | 4.1.0 | 4.1.0+computecanada |
| six | 1.16.0 | 1.16.0+computecanada |
| smmap | 5.0.1 | 5.0.1+computecanada |
| sniffio | 1.3.0 | 1.3.0+computecanada |
| soupsieve | 2.4.1 | 2.4.1+computecanada |
| stack_data | 0.6.2 | 0.6.2+computecanada |
| sympy | 1.12 | 1.12+computecanada |
| terminado | 0.17.1 | 0.17.1+computecanada |
| testpath | 0.6.0 | 0.6.0+computecanada |
| threadpoolctl | 3.3.0 | 3.3.0+computecanada |
| tinycss2 | 1.2.1 | 1.2.1+computecanada |
| tokenizers | 0.15.0 | 0.15.0+computecanada |
| torch | N/A | 2.2.1+computecanada |
| tornado | 6.3.3 | 6.3.3+computecanada |
| tqdm | 4.66.2 | 4.66.2+computecanada |
| traitlets | 5.9.0 | 5.9.0+computecanada |
| transformers | 4.38.1 | 4.38.1+computecanada |
| typing_extensions | 4.10.0 | 4.10.0+computecanada |
| tzdata | 2023.3 | 2023.3+computecanada |
| urllib3 | 2.0.4 | 2.0.4+computecanada |
| wandb | 0.16.0 | 0.16.0+computecanada |
| wcwidth | 0.2.6 | 0.2.6+computecanada |
| webencodings | 0.5.1 | 0.5.1+computecanada |
| websocket_client | 1.6.2 | 1.6.2+computecanada |
| xxhash | 3.2.0 | 3.2.0+computecanada |
| yarl | 1.9.3 | 1.9.3+computecanada |
| zipp | 3.16.2 | 3.16.2+computecanada |

# Table S5. Performance of four explainers (SHAP, IG, GPT-index, GPT-token) based on mean area over the perturbation curve (AOPC) across 141 correctly classified stratified articles sampled from the McMaster PLUS and Clinical Hedges databases (2003 to 2024), classified for methodological rigour using a fine-tuned BioLinkBERT model. Higher AOPC indicates greater attribution faithfulness.

| *Explainer* | *AOPC (All Tokens)* | *AOPC (Tokens with Positive Attributions)* | *AOPC (Tokens with Negative Attributions)* |
| --- | --- | --- | --- |
| SHAP | 0.212 (0.184, 0.240) | 0.255 (0.219, 0.291) | 0.039 (0.031, 0.047) |
| IG | 0.214 (0.185, 0.243) | 0.303 (0.260, 0.346) | 0.024 (0.016, 0.032) |
| GPT-index | 0.033 (0.017, 0.049) | 0.046 (0.025, 0.068) | -0.010 (-0.022, 0.003) |
| GPT-token | 0.024 (0.007, 0.042) | 0.041 (0.017, 0.066) | -0.015 (-0.028, -0.003) |

**AOPC** Area over the perturbation curve; **GPT** Generative Pretrained Transformer; **IG** Integrated gradient; **SHAP** SHapley Additive exPlanations.

*Note:* All values are shown as the mean (95% CI) across the 141 instances.

# **Table S6.** Pairwise correlation and distribution similarity of token-level feature attributions generated by four explainers (SHAP partition explainer, integrated gradients, GPT-index, and GPT-token) across 57,195 tokens from the 141 correctly classified stratified articles sampled from the McMaster PLUS and Clinical Hedges databases (2003 to 2024), classified for methodological rigour using a fine-tuned BioLinkBERT model. Pearson's r, Spearman's ρ, and Kendall's τ assess linear and rank-based correlation; Wasserstein distance measures distributional similarity between attribution value distributions.

| *Explainer A* | *Explainer B* | *Pearson’s r* | *Spearman’s ρ* | *Kendall’s τ* | *Wasserstein Distance* |
| --- | --- | --- | --- | --- | --- |
| SHAP | IG | 0.358* | 0.293* | 0.205* | 0.002 |
| SHAP | GPT-index | -0.048* | 0.077* | 0.050* | 0.005 |
| SHAP | GPT-token | 0.003 | 0.041* | 0.027* | 0.004 |
| IG | GPT-index | 0.011* | 0.050* | 0.034* | 0.006 |
| IG | GPT-token | 0.024* | 0.028* | 0.019* | 0.005 |
| GPT-index | GPT-token | 0.099* | 0.119* | 0.088* | 0.001 |

*Statistical significance (P<0.05).

**GPT** Generative Pretrained Transformer; **IG** Integrated gradient; **SHAP** SHapley Additive exPlanations.

# Figure S1. Accumulated local feature attributions of the identified most important negative and positive tokens with ≥1 occurrences generated by four explainers (SHAP partition explainer, integrated gradients, GPT-index, and GPT-token) across 80,901 tokens from 200 stratified articles sampled from the McMaster PLUS and Clinical Hedges databases (2003 to 2024). Values are mean and 95% CI.


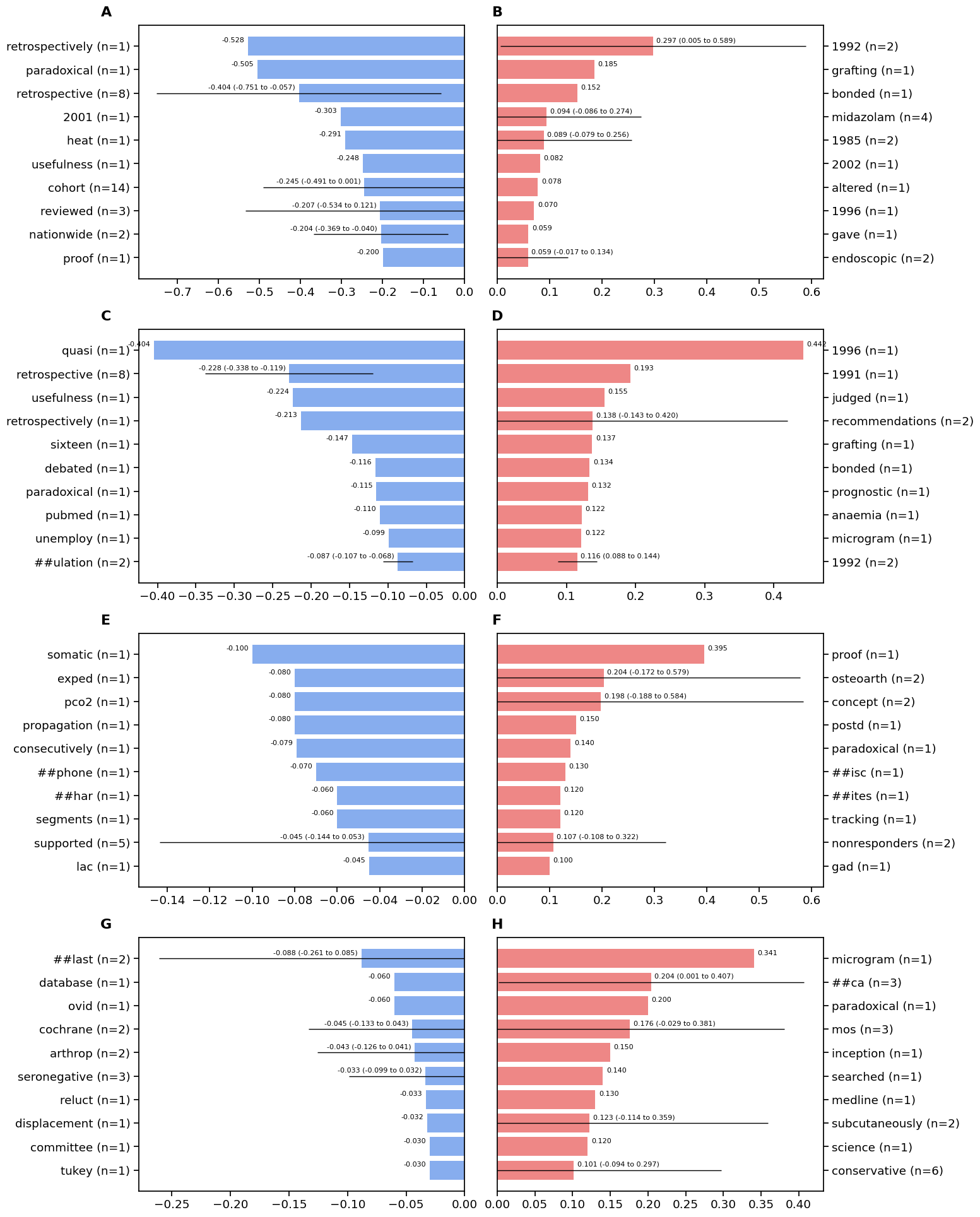


**A** Negative tokens for SHAP; **B** Positive tokens for SHAP; **C** Negative tokens for IG; **D** Positive tokens for IG; **E** Negative tokens for GPT-index; **F** Positive tokens for GPT-index; **G** Negative tokens for GPT-token; **H** Positive tokens for GPT-token.

# Figure S2. Accumulated local feature attributions of the identified most important negative and positive tokens with ≥100 occurrences generated by four explainers (SHAP partition explainer, integrated gradients, GPT-index, and GPT-token) across 80,901 tokens from 200 stratified articles sampled from the McMaster PLUS and Clinical Hedges databases (2003 to 2024). Values are mean and 95% CI.


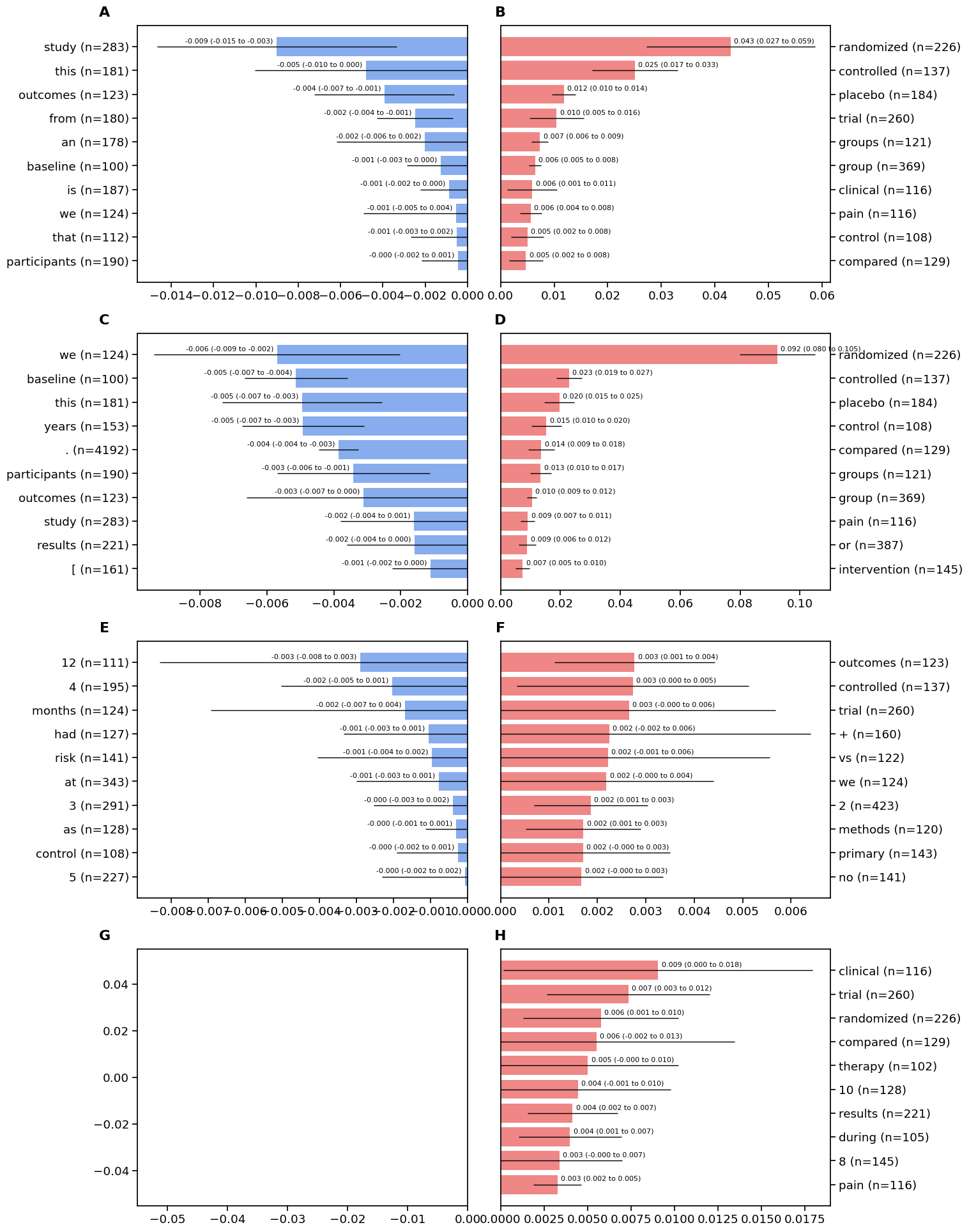


**A** Negative tokens for SHAP; **B** Positive tokens for SHAP; **C** Negative tokens for IG; **D** Positive tokens for IG; **E** Negative tokens for GPT-index; **F** Positive tokens for GPT-index; **G** Negative tokens for GPT-token; **H** Positive tokens for GPT-token.

# Figure S3. Perturbation curves of the four explainers (SHAP, IG, GPT-index, GPT-token) across 141 correctly classified stratified articles sampled from the McMaster PLUS and Clinical Hedges databases (2003 to 2024), classified for methodological rigour using a fine-tuned BioLinkBERT model. Shaded areas represent the 95% CI.


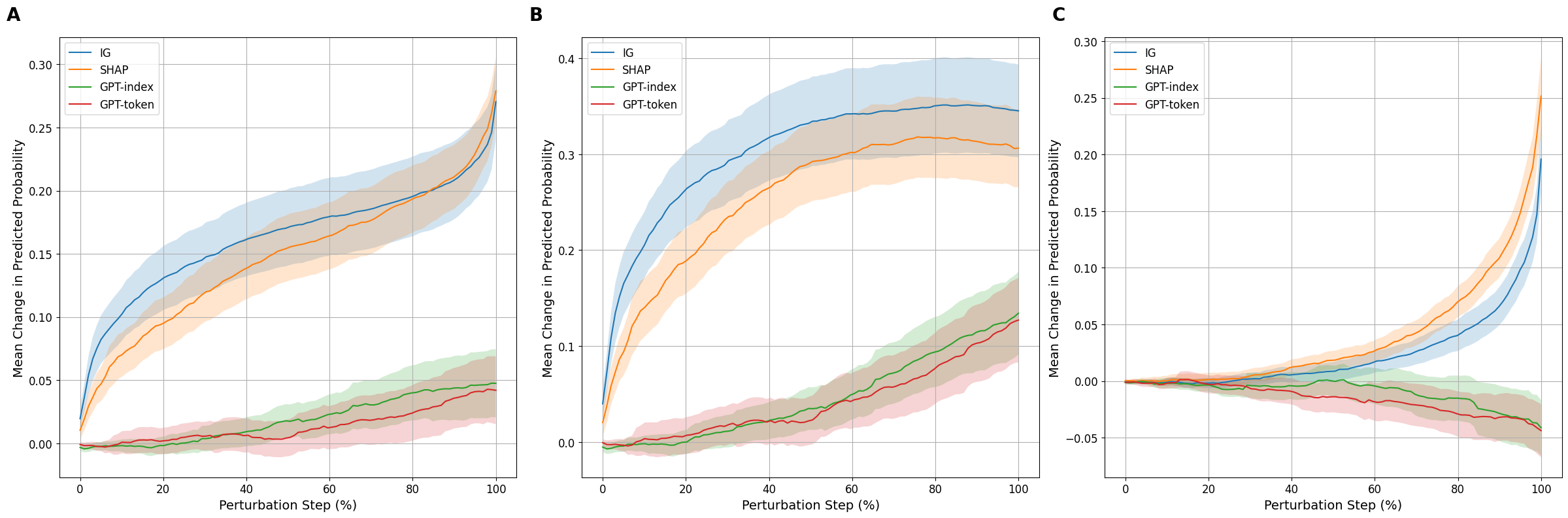


**A** All tokens; **B** Tokens with positive attributions; **C** Tokens with negative attributions.

**GPT** Generative Pretrained Transformer; **IG** Integrated gradient; **SHAP** SHapley Additive exPlanations.

# Figure S4. Scatter plots of token-level feature attributions generated by four explainers (SHAP partition explainer, integrated gradients, GPT-index, and GPT-token) across 57,195 tokens from 141 correctly classified stratified articles sampled from the McMaster PLUS and Clinical Hedges databases (2003 to 2024), classified for methodological rigour using a fine-tuned BioLinkBERT model.


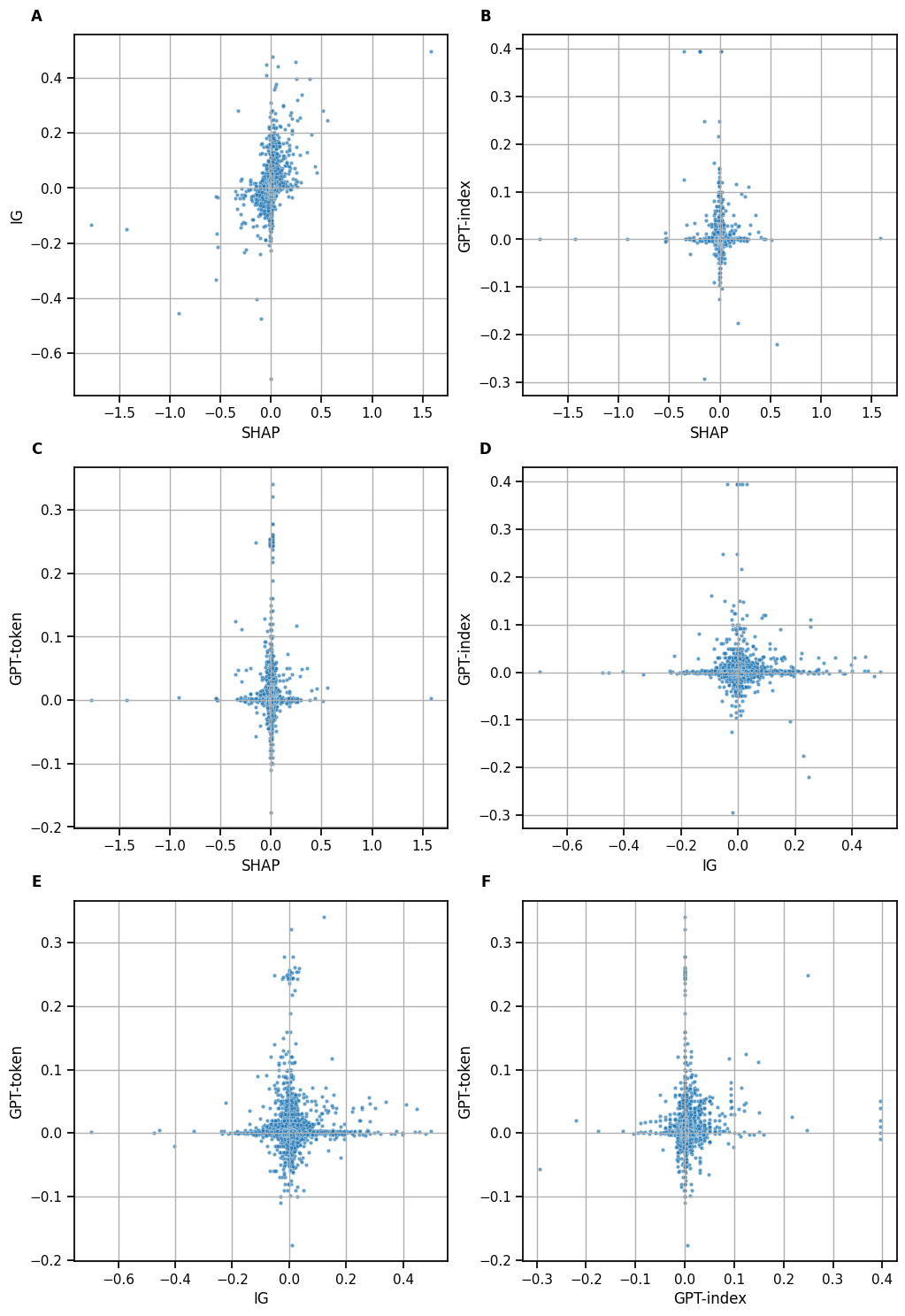


**A** SHAP and IG; **B** SHAP and GPT-index; **C** SHAP and GPT-token; **D** IG and GPT-index; **E** IG and GPT-token; **F** GPT-index and GPT-token.

# Figure S5. Accumulated local feature attributions of the identified most important negative and positive tokens with ≥1 occurrences generated by four explainers (SHAP partition explainer, integrated gradients, GPT-index, and GPT-token) across 57,195 tokens from 141 correctly classified stratified articles sampled from the McMaster PLUS and Clinical Hedges databases (2003 to 2024). Values are mean and 95% CI.


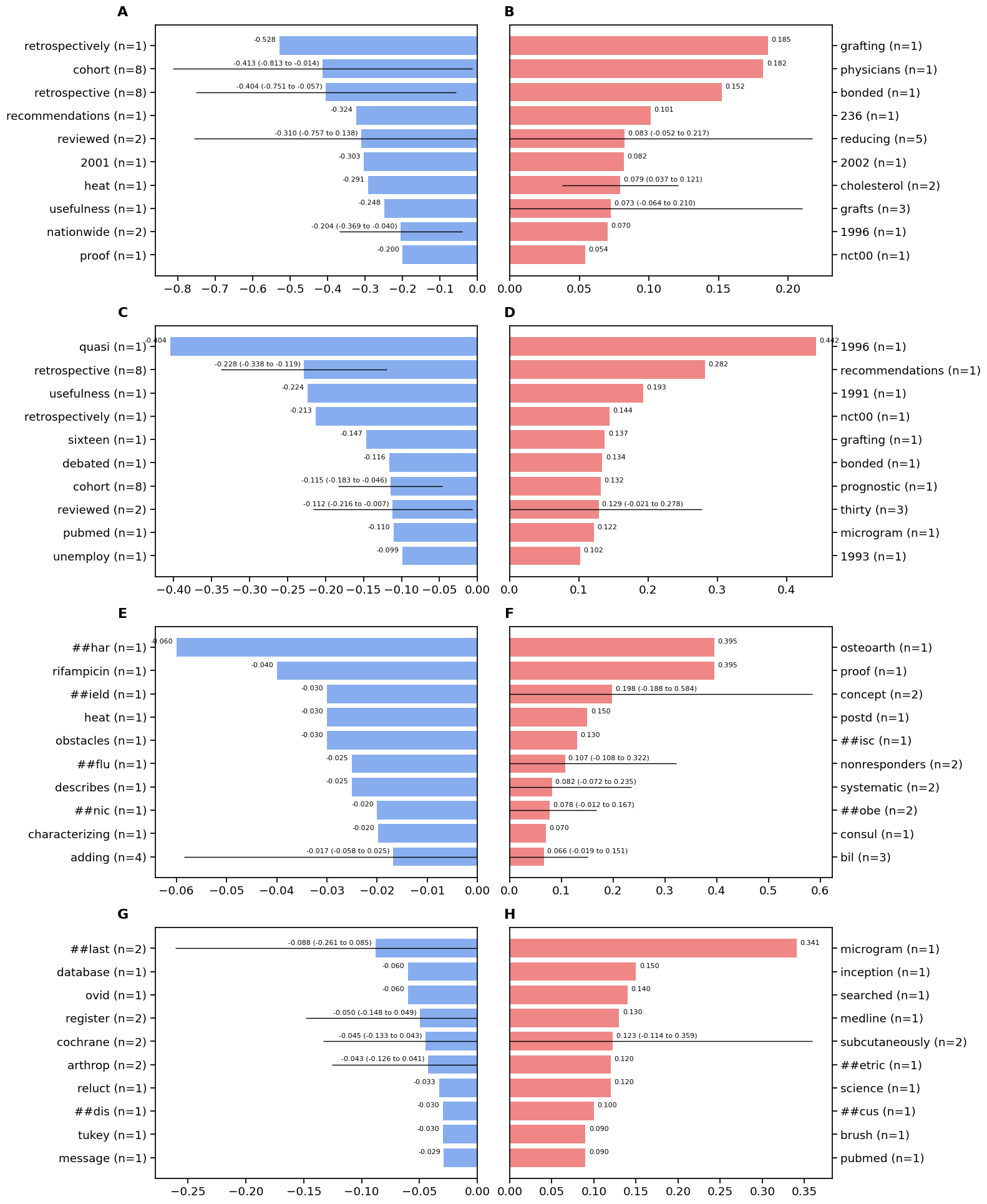


**A** Negative tokens for SHAP; **B** Positive tokens for SHAP; **C** Negative tokens for IG; **D** Positive tokens for IG; **E** Negative tokens for GPT-index; **F** Positive tokens for GPT-index; **G** Negative tokens for GPT-token; **H** Positive tokens for GPT-token.

# Figure S6. Accumulated local feature attributions of the identified most important negative and positive tokens with ≥10 occurrences generated by four explainers (SHAP partition explainer, integrated gradients, GPT-index, and GPT-token) across 57,195 tokens from 141 correctly classified stratified articles sampled from the McMaster PLUS and Clinical Hedges databases (2003 to 2024). Values are mean and 95% CI.


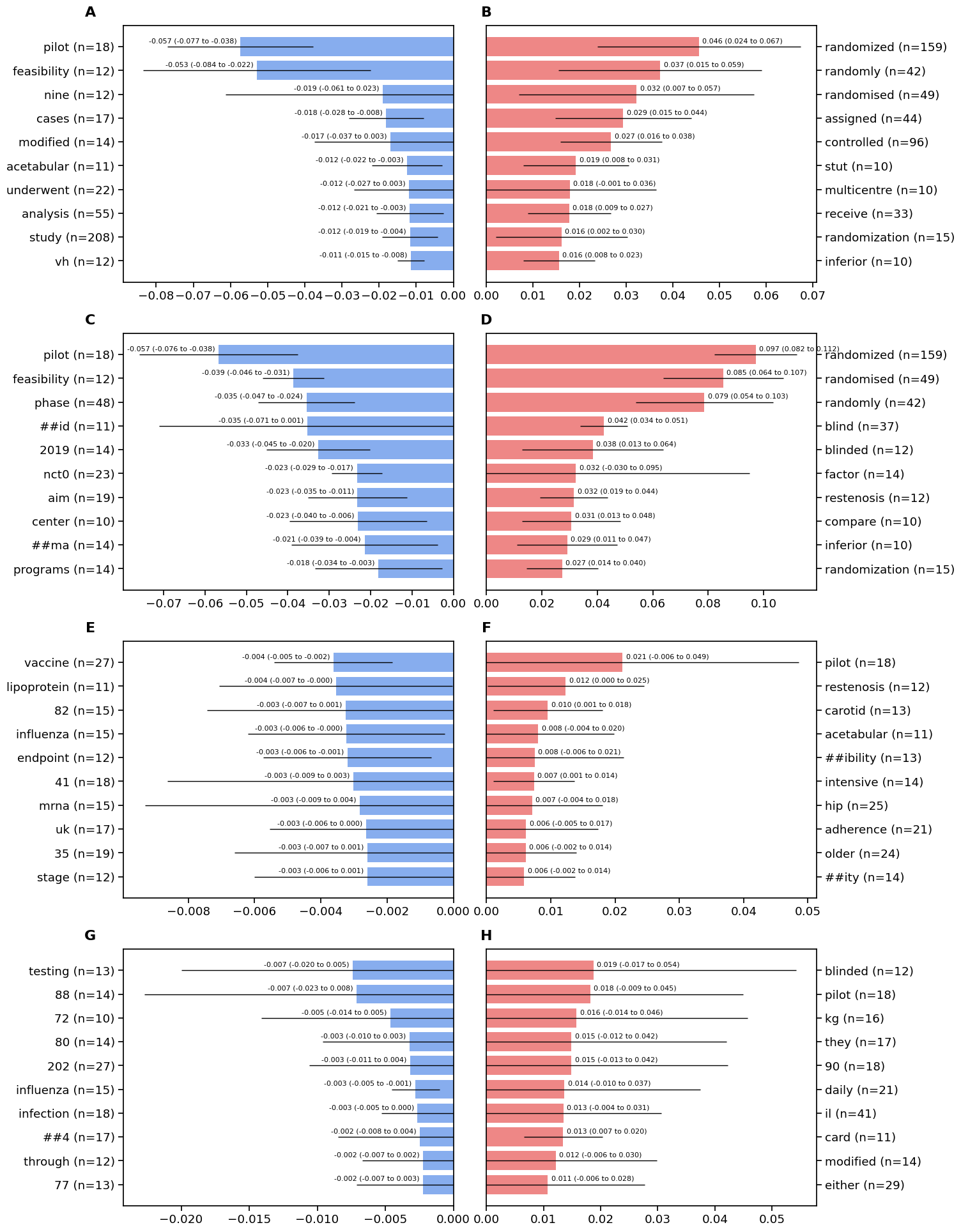


**A** Negative tokens for SHAP; **B** Positive tokens for SHAP; **C** Negative tokens for IG; **D** Positive tokens for IG; **E** Negative tokens for GPT-index; **F** Positive tokens for GPT-index; **G** Negative tokens for GPT-token; **H** Positive tokens for GPT-token.

**Figure S7.** Accumulated local feature attributions of the identified most important negative and positive tokens with ≥100 occurrences generated by four explainers (SHAP partition explainer, integrated gradients, GPT-index, and GPT-token) across 57,195 tokens from 141 correctly classified stratified articles sampled from the McMaster PLUS and Clinical Hedges databases (2003 to 2024). Values are mean and 95% CI.
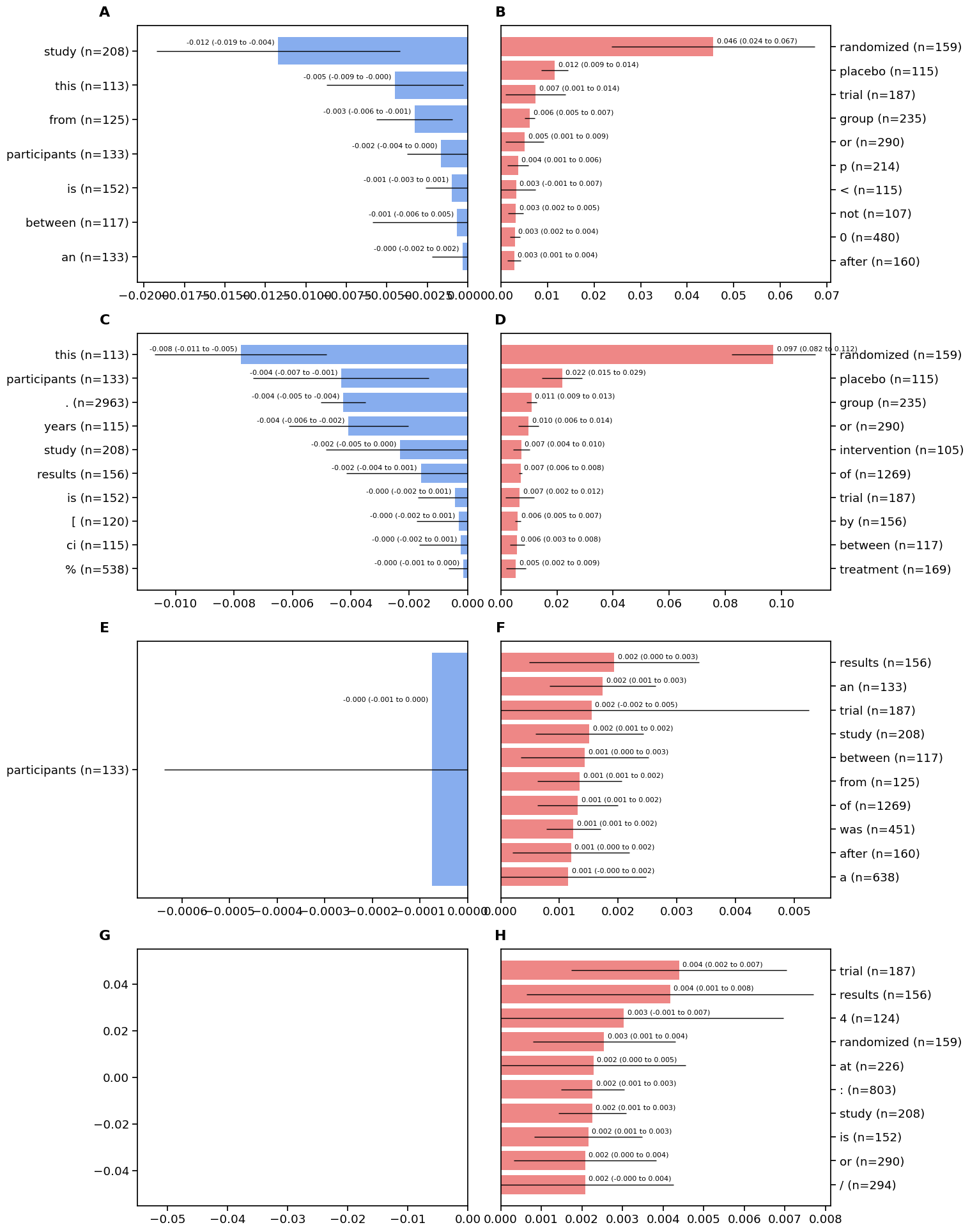


**A** Negative tokens for SHAP; **B** Positive tokens for SHAP; **C** Negative tokens for IG; **D** Positive tokens for IG; **E** Negative tokens for GPT-index; **F** Positive tokens for GPT-index; **G** Negative tokens for GPT-token; **H** Positive tokens for GPT-token.
